# A biocompatible stapling reaction for *in situ* generation of constrained peptides†

**DOI:** 10.1039/d0sc05125j

**Published:** 2020-11-04

**Authors:** Richard Morewood, Christoph Nitsche

**Affiliations:** Research School of Chemistry, Australian National University Canberra ACT 2601 Australia christoph.nitsche@anu.edu.au

## Abstract

Constrained peptides are promising next-generation therapeutics. Peptide stapling is a particularly attractive technique to generate constrained macrocycles with improved biological activity and metabolic stability. We introduce a biocompatible two-component stapling approach based on the reagent 2,6-dicyanopyridine and a pseudo-cysteine amino acid. Stapling can proceed either directly on-resin during solid-phase synthesis or following isolation of the linear peptide. The stapling reaction is orthogonal to natural amino acid side chains and completes in aqueous solution at physiological pH, enabling its direct use in biochemical assays. We performed a small screening campaign of short peptides targeting the Zika virus protease NS2B-NS3, allowing the direct comparison of linear with *in situ* stapled peptides. A stapled screening hit showed over 28-fold stronger inhibition than its linear analogue, demonstrating the successful identification of constrained peptide inhibitors.

## Abstract 2

A synthetically straightforward and biocompatible peptide-stapling strategy that can be used directly in biochemical assays to identify constrained enzyme inhibitors.

## Graphical abstract

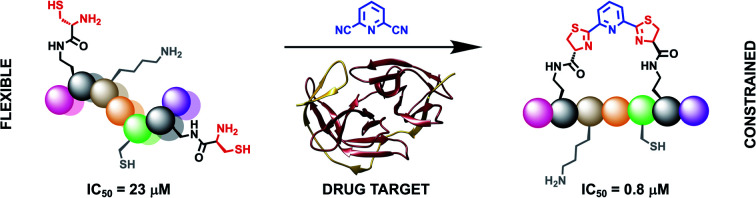

## Introduction

**Scheme 1:**
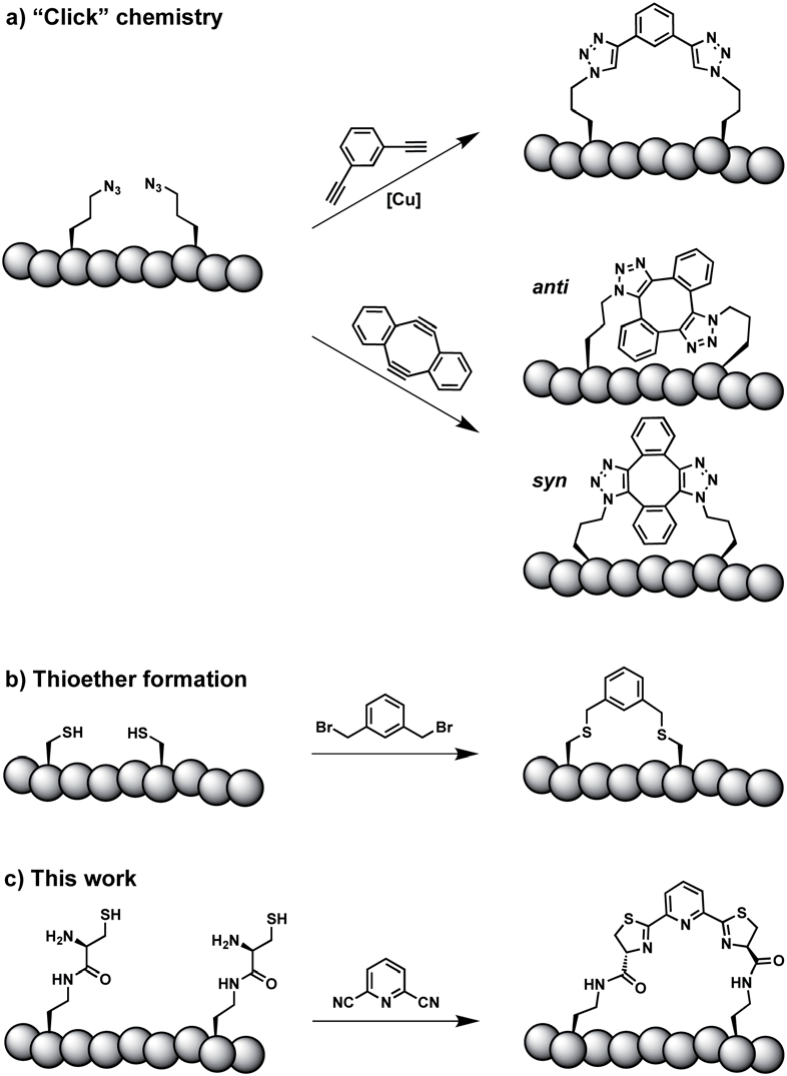
Two-component peptide stapling strategies. (a) Copper-catalysed azide–alkyne cycloaddition and strain-promoted azide–alkyne cycloaddition (SPAAC). (b) Cysteine conjugation. (c) Thiazoline formation presented in this study.

Constrained peptides are considered to combine the best attributes of antibodies and small molecules, positioning them well as promising next-generation therapeutics.^1^ Constrained structures can be engineered *via* intramolecular covalent bonds in order to improve the bio- and physicochemical properties.^2^ Peptide stapling is a particularly straightforward technique to trigger conformational constraint in peptides.^3^ Two-component stapling strategies use reagents that specifically react with two amino acid side chains in linear peptides.^4^ Established approaches capitalize, for example, on azide–alkyne “Click” chemistry or conjugation of cysteine residues (Scheme 1).^5^

Here we report an alternative two-component stapling strategy that can be used directly in biochemical set-ups, such as enzymatic assays. Conventional stapling approaches are often not fully biocompatible, preventing their use in presence of proteins. Copper catalysts used in “Click” chemistry can result in protein precipitation,^6^ while cysteine-reactive staples obviously suffer from incompatibilities with natural cysteine residues. Double strain-promoted azide–alkyne cycloaddition (SPAAC) is a biocompatible variant of “Click” chemistry that unfortunately suffers from large hydrophobic staples and *syn* and *anti* regioisomerism (Scheme 1).^7^ Water-soluble staples used in SPAAC are permanently charged and require laborious chemical synthesis.^8^

The presented approach overcomes previous limitations, building on the biocompatible reaction between 1,2-aminothiols and 2-cyanopyridine,^9^ which is fully orthogonal to all canonical amino acids and does not require any catalysts. Peptides containing 1,2-aminothiol functional groups are assembled from standard building blocks during solid-phase synthesis and stapled with commercially available 2,6-dicyanopyridine (DCP). Compared to SPAAC, the stapling reaction is regioselective and the resulting linker is smaller and less hydrophobic (Scheme 1).

In contrast to alkyl or benzyl nitriles,^10^ (hetero)aryl nitriles like DCP do not represent a source of toxic cyanide. Nitrile hydrolysis to amides occurs only under extreme pH conditions or very high temperature.^11^ Consequently, DCP is a non-toxic, water-stable and thus biocompatible stapling reagent.

## Results and discussion

### Stapling strategy

**Scheme 2:**
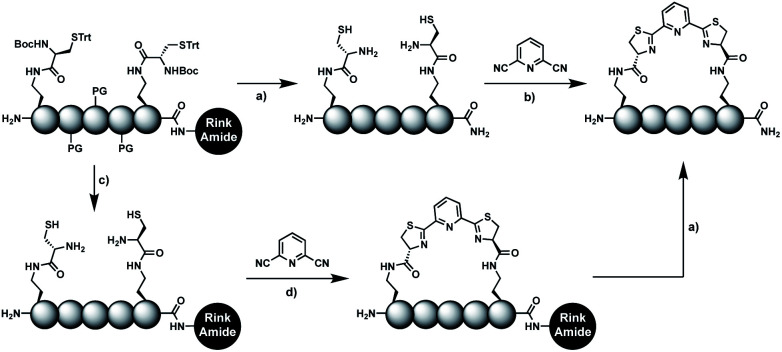
Solution- and solid-phase stapling of peptides using the pseudo-cysteine amino acid Dys and 2,6-dicyanopyridine (DCP). PG indicates standard side-chain protection groups. (a) TFA/TIPS/EDT/H_2_O (91 : 3 : 3 : 3), 2 h. (b) Tris pH 7.5, TCEP. (c) DCM/TFA/TIPS/EDT (65 : 25 : 5 : 5), 2 × 3 min. (d) DMF, DIPEA, 2 h.

In order to introduce the 1,2-aminothiol functional group in peptide side chains,^12^ we coupled l-2,4-diaminobutyric acid (Dab) to l-cysteine (Cys) to create the pseudo-cysteine amino acid Dab(Cys), which we refer to as Dys. We synthesized Fmoc-Dys(Boc,Trt)-OH (20) (Scheme S1†), which is fully compatible with solid-phase peptide synthesis, and introduced commercially available ($ 5 per g) DCP as reagent to staple linear peptides containing two Dys residues *via* double thiazoline formation (Scheme 2). Peptides can be stapled either after release from the solid support or directly on the resin (Scheme 2).

### Solution-phase stapling

**Fig. 1:**
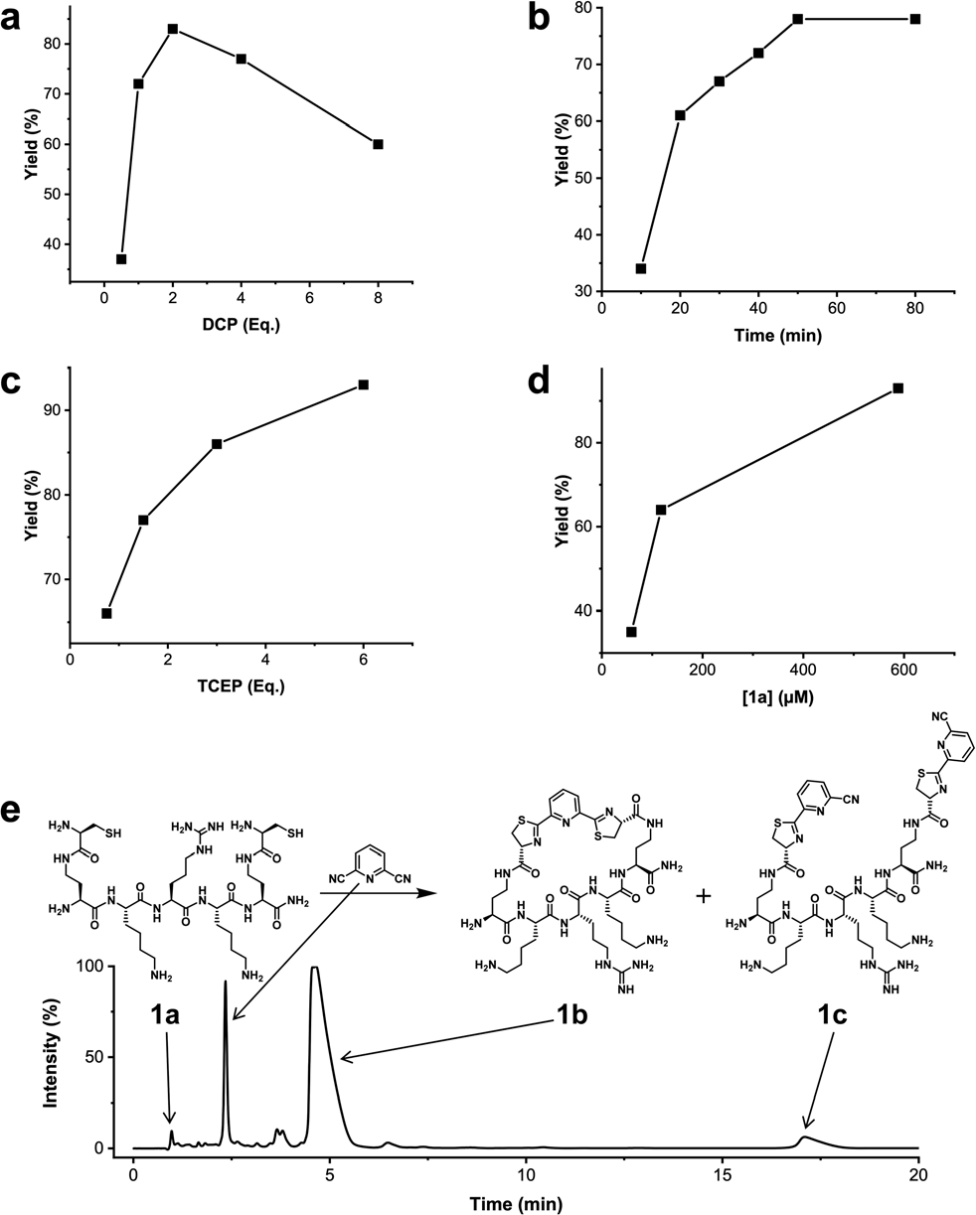
Analysis of reaction parameters in the synthesis of 1b from 1a and the staple 2,6-dicyanopyridine (DCP) in 10 mM Tris pH 7.5. The yields reported refer to 1b. (a) 0.6 mM 1a, 0.9 mM TCEP. (b) 0.6 mM 1a, 1.2 mM DCP, 0.9 mM TCEP. (c) 0.6 mM 1a, 1.2 mM DCP. (d) DCP (2 Eq.), TCEP (6 Eq.). (e) LC-MS (254 nm, method A) using optimized conditions: 0.6 mM 1a, 1.2 mM DCP, 3.5 mM TCEP, 1 h.

We analysed the *in situ* stapling conditions in solution for the model compound 1a (H-Dys-Lys-Arg-Lys-Dys-NH_2_) in detail to identify optimal reaction parameters (Fig. 1). To reflect physiological conditions as best as possible, we operated at pH 7.5 during all experiments. We observed highest yields for cyclic peptide 1b using up to 2 equivalents of DCP (Fig. 1a). Ratios of DCP : 1a exceeding 2 resulted in a gradual decrease of 1b in favour of the double DCP-capped by-product 1c. However, even in presence of an 8-fold excess of DCP, the overall yield of 1b was still 60%, indicating a strongly favoured cyclic product. The reaction completes in less than 1 h at 0.6 mM 1a (Fig. 1b). As expected for a bimolecular reaction, the reaction rate is concentration-dependent (Fig. 1d). If exposed to air, 1a has a strong tendency to form a cyclic disulfide, which is unreactive with DCP. It is therefore important to perform the cyclisation reaction in presence of the reducing agent TCEP. Up to 6 equivalents of TCEP are necessary for high yield (Fig. 1c). Under these optimized parameters, we were able to improve the overall yield of 1b to 93%, as determined by LC-MS using a standard curve of pure 1b.

### Solid-phase stapling

**Fig. 2:**
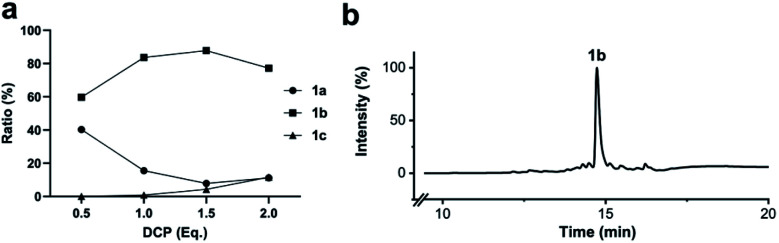
Optimization of solid-phase synthesis of compound 1b on Rink Amide resin. (a) Ratios of linear peptide 1a, stapled peptide 1b and double DCP-capped by-product 1c as functions of the 2,6-dicyanopyridine (DCP) content. (b) LC-MS (254 nm, method B) of crude 1b after cleavage from the solid support.

To simplify the synthesis of constrained peptides even further, we developed a method to staple the linear peptide directly on the solid support. In order to minimize potential dimerization reactions, we used a low-loading resin (0.38 mmol g^−1^). Short exposure to 25% TFA deprotected peptide 1a without releasing it from the Rink Amide resin (Scheme 2). Subsequent treatment with DCP and DIPEA in DMF stapled the peptide on the solid phase. Standard cleavage conditions using over 90% TFA released the fully intact cyclic peptide 1b. Analysing the ratio of linear peptide 1a, cyclic peptide 1b and double DCP-capped by-product 1c suggested that 1.5 equivalents of DCP are optimal for on-resin stapling (Fig. 2a). Less DCP resulted in more unreacted 1a, while a larger DCP excess increased by-product 1c. The optimized on-resin stapling conditions resulted in high purity of 1b directly after cleavage (Fig. 2b). Remarkably, the 2 hour cleavage procedure in presence of over 90% TFA caused only very minor hydrolysis of the thiazoline heterocycles (<5%). However, after 1 h, we observed full hydrolysis of both thiazolines in water containing 2% TFA and were able to isolate and characterize the hydrolysis product 1d (Scheme S2†). We never observed hydrolysis in water containing 0.1% TFA (pH 2.0) or 0.1% formic acid (pH 2.7), both frequently used during peptide purification (data not shown).

### Scope of the stapling strategy

In order to use a peptide stapling strategy *in situ* while screening for constrained peptide inhibitors, the chemical approach not only needs to be biocompatible, but also synthetically robust with respect to the formation of the desired product. For example, polymerization in favour of cyclization would significantly interfere with biochemical assays. To address this concern, we synthesized 17 additional linear peptides 2a-18a, all of different length and sequence (Table 1).

**Table 1:** Peptides stapled using 2,6-dicyanopyridine (DCP) and pseudo-cysteine amino acids

Cpd^a^	Sequence^b^	Yield^c^ (%)
1b	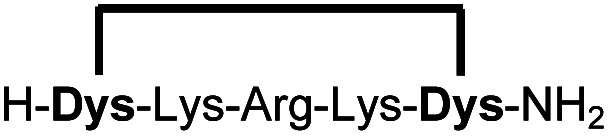	89 (93)^d^
2b	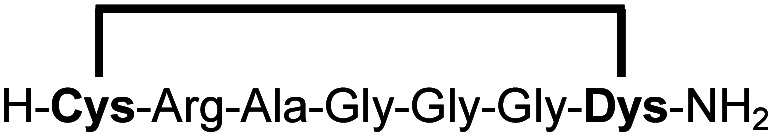	87
3b	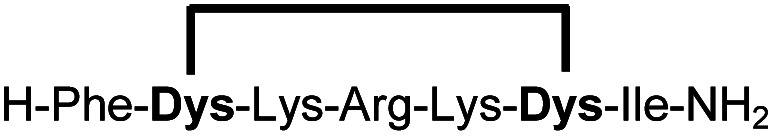	93
4b	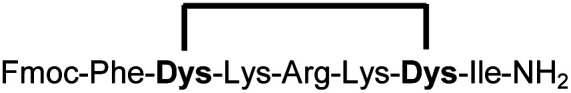	90
5b	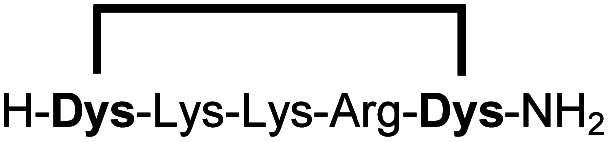	96
6b	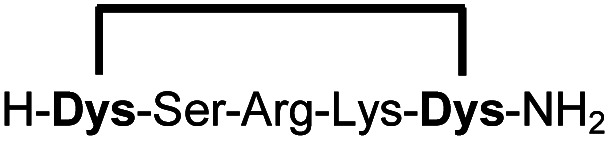	90
7b	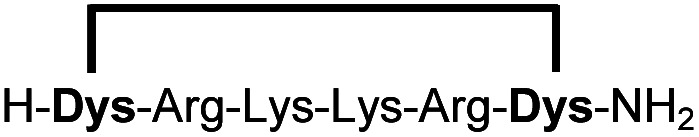	95
8b	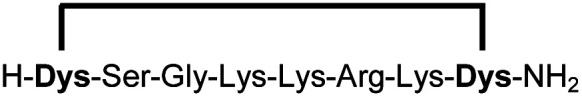	97
9b	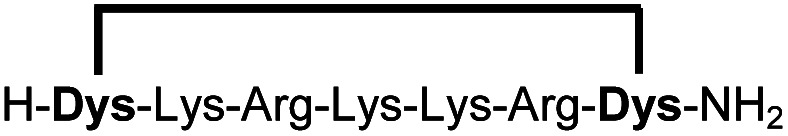	94
10b	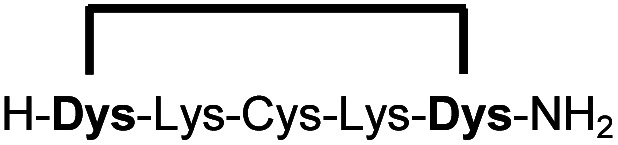	90
11b	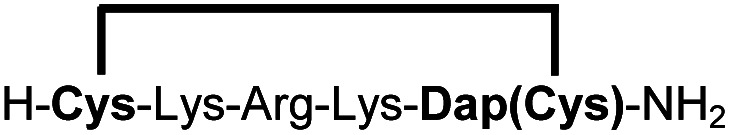	83^e^
12b	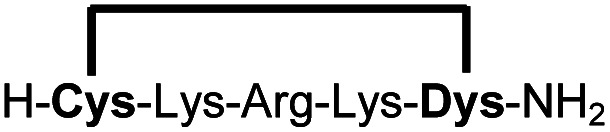	76^e^
13b	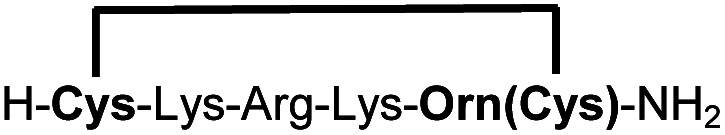	79^e^
14b	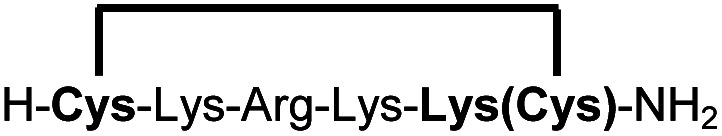	83^e^
15b	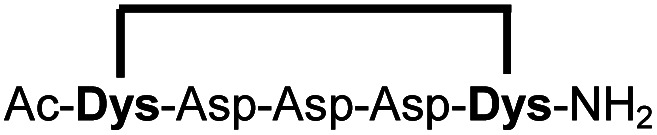	87
16b	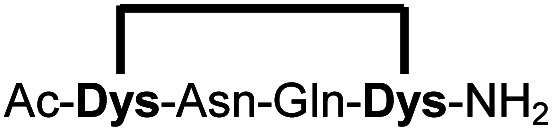	95
17b	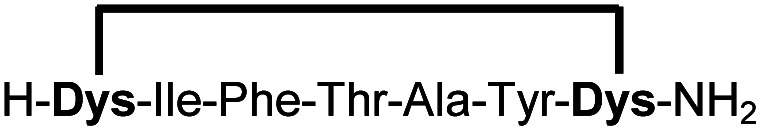	83^f^
18b	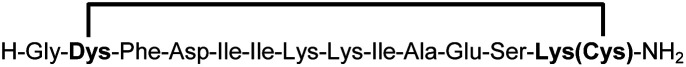	86

a Macrocyclic peptides stapled from their linear precursors 1a-18a.

b Dys = Dab(Cys).

c Yield determined by LC-MS (254 nm, method B) after 24 h incubation of 0.6 mM linear peptides 1a-18a with 1.2 mM 2,6-dicyanopyridine (DCP) in 10 mM Tris pH 7.5, 3.5 mM TCEP.

d Yield after 1 h of incubation (254 nm, method A).

e Yield after 6 h of incubation.

f Stapled in 10 mM Tris pH 7.5, 3.5 mM TCEP, 75% MeOH (v/v).

Linear peptides 1a-10a and 15a-18a were stapled using the optimized conditions and analysed by LC-MS after 24 h (11a-14a were analysed after 6 h). We deliberately chose a long incubation time to ensure reaction completion for all sequences and to assess the stability in buffer over a longer period of time. We observed yields ranging from 76% to 97% for all stapled peptides 1b-18b, clearly indicating the robustness and reliability of the presented stapling methodology. All sequence variations between two pseudo-cysteine residues were well tolerated (Table 1). Hydrophobic residues exceeding both Dys amino acids at the N- and C-termini (3b, 4b) had no impact on the stapling yield. In case of compounds 7b-9b and 16b we detected additional minor species of identical molecular mass (Fig. S29–S31 and S38†), potentially indicating topological isomers.

To prove that the stapling is fully orthogonal to all canonical amino acids, we included a central cysteine residue in the sequence of peptide 10a, which was stapled to compound 10b in exceptional yield of 90%. The investigated compounds cover all reactive groups usually present in peptides and proteins, such as alcohols (Ser), phenols (Tyr), thiols (Cys), amines (Lys) and guanidines (Arg), none of which showed any cross-reactivity with the DCP staple at pH 7.5 during the 24 h incubation period.

The shortest peptide investigated (16a) contains only two amino acids between both pseudo-cysteine residues, whilst the longest peptide has ten residues between them (18a). Both peptides were successfully stapled in high yield, clearly indicating the broad scope of the presented approach.

Peptides 1b-14b all contain at least one positively charged residue. To further demonstrate that the presented strategy is independent on the peptide substrate, we synthesized a strongly negatively charged cyclic peptide 15b and an uncharged cyclic peptide 16b. We further investigated a very hydrophobic peptide (17a), which was also stapled in high yield. However, due to insufficient water solubility of 17b at 0.6 mM, stapling had to proceed in buffer containing 75% methanol.

To further expand the scope towards longer naturally occurring peptides, we synthesized the 13 amino acid long antibacterial and anticancer active peptide aurein 1.2, which is secreted from the granular dorsal glands of the green and golden bell frog *Litoria aurea*.^13^ We positioned two pseudo-cysteine residues in *i*, *i* + 11 of aurein 1.2 derivative 18a and stapled it to 18b in 86% yield.

### Solid-phase assembly of different pseudo-cysteine residues

**Scheme 3:**
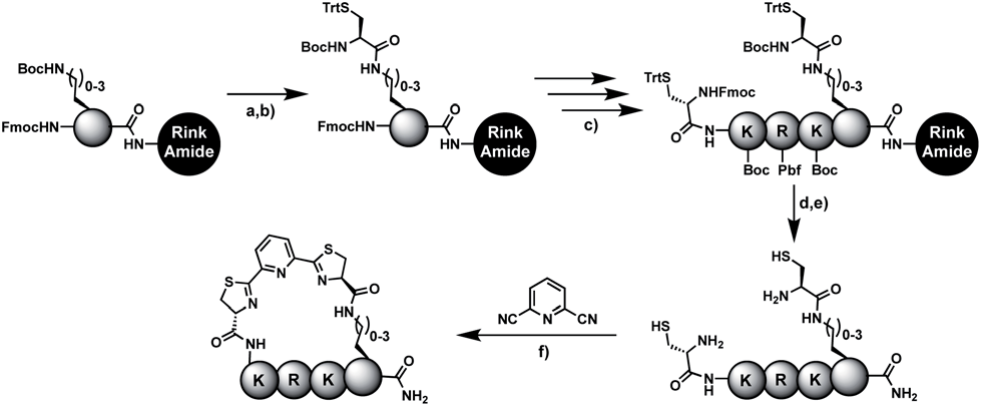
Solid-phase assembly of pseudo cysteines with varying linker length and subsequent stapling with 2,6-dicyanopyridne to constrained peptides 11b-14b. (a) DCM/TFA (75 : 25), 2 × 3 min. (b) Boc-Cys(Trt)-OH, HBTU, HOBt, DIPEA, DMF, 1 h. (c) Standard Fmoc solid-phase peptide synthesis in the order Fmoc-Lys(Boc)-OH, Fmoc-Arg(Pbf)-OH, Fmoc-Lys(Boc)-OH, Fmoc-Cys(Trt)-OH. (d) piperidine, DMF. (e) TFA/TIPS/EDT/H_2_O (91 : 3 : 3 : 3), 2 h. (f) Tris pH 7.5, TCEP, 6 h.

Biocompatible stapling reactions offer direct applications in drug discovery. However, in order to be broadly applicable, the chemical approach needs to be straightforward. Ideally, the synthesis can be fully automated to (i) make it amendable to researchers with limited access to synthetic chemistry, (ii) facilitate affordable synthesis on demand and (iii) allow for the generation of large screening libraries. To address this demand, we developed an alternative synthetic route to peptides bearing two 1,2-aminothiol groups, which is fully compatible with automated solid-phase peptide synthesis (Scheme 3). It involves the on-resin assembly of a C-terminal pseudo-cysteine residue, followed by the synthesis of the desired peptide sequence which is concluded by an N-terminal cysteine residue (as in 2a).

We explored different linker lengths for pseudo cysteine to further expand the chemistry toolbox for the generation of constrained peptides (Scheme 3). Linkers can be varied by employing standard-protected variants of l-2,3-diaminopropionic acid (Dap), l-2,4-diaminobutyric acid (Dab), ornithine (Orn) and lysine (Lys). Following this approach, we synthesized peptides 11a-14a comprising the core sequence Lys-Arg-Lys (Scheme 3). All peptides were fully assembled on solid support, before release and stapling with DCP to furnish 11b-14b in yields ranging from 76% to 83% (Scheme 3). The slightly decreased stapling yields compared to 1b-10b and 15b-18b might be an indication of pronounced strain in these macrocycles. This conclusion is supported by the significant formation of the double DCP-capped by-products 11c-14c with yields between 12% and 16% (Fig. S33–S36†).

A similar strategy was applied for the synthesis of aurein 1.2 derivative 18a, where a hybrid strategy with two different pseudo-cysteine residues was chosen. Lys(Cys) was installed at the C-terminus of aurein 1.2 using solid-phase assembly and a Dys residue was incorporated at position 2 using the standard procedure. This demonstrates the installation of two pseudo-cysteine residues with different linker lengths in the same peptide. Combining two of the four introduced pseudo cysteines allows for up to 16 different staple geometries in any constrained peptide of interest.

### Screening campaign against Zika virus NS2B-NS3 protease

High stapling yields directly relate to compound purity sufficient for inhibitor screening campaigns, particularly if the excess of stapling reagent can be considered inert with respect to biochemical systems. Therefore, we set out to demonstrate that the *in situ* generation of constrained peptides, using the chemistry presented, can be employed for screening campaigns. Linear peptides 1a-10a were designed as a series of potential inhibitors targeting the protease of Zika and related flaviviruses.^14^ The protease NS2B-NS3 of Zika virus (ZiPro) is involved in processing the viral polyprotein and thus a replication-essential enzyme and promising drug target. ZiPro recognizes peptide sequences which comprise at least two basic residues (Arg or Lys). Consequently, at least one basic amino acid is present in compounds 1a-10a. As recently demonstrated for inhibitors of NS2B-NS3 from Zika and West Nile viruses, cyclisation of peptide substrates can guard against proteolysis and boost affinity by lowering the entropic penalty associated with the binding process.^9,15^ Consequently, ZiPro is an excellent target to conduct a small screening campaign of linear and constrained peptides using the presented stapling strategy.

We screened linear peptides 1a-10a for ZiPro inhibition at two concentrations of 10 and 1 μM (Table 2). In order to compare biological activities directly with their stapled analogues, we incubated peptides 1a-10a with DCP and screened the *in situ* stapled peptides 1b-10b at identical concentrations (Table 2). This experiment was conducted without any additional purification step between stapling and screening. Some stapled peptides showed increased (1b, 2b, 3b, 6b) others decreased inhibition (4b, 5b, 7b) compared to their linear analogues. Two pairs (8a/8b, 9a/9b) displayed only insignificant differences. It is not surprising that some stapled peptides display reduced binding affinity compared to their linear analogues. Stapling limits the conformational flexibility of the peptide substrate and may thus hinder or even prevent formation of the active conformation. However, if the peptide is constrained in its most active state, stapling may increase affinity. In our ZiPro screening campaign this effect was most pronounced for peptide 1b, which showed a very strong increase of inhibition at both tested concentrations compared to its linear analogue 1a (Table 2). We therefore decided to investigate the pair 1a/1b in more detail.

**Table 2:** Results of the Zika virus NS2B-NS3 protease (ZiPro) inhibition assay

Cpd^a^	ZiPro inhibition^c^ (%)		Cpd^b^	ZiPro inhibition^c^ (%)	
	10 μM	1 μM		10 μM	1 μM
1a	23 ± 4	n.i.	1b	60 ± 7	27 ± 4
2a	n.i.	n.i.	2b	19 ± 1	n.i.
3a	35 ± 3	11 ± 4	3b	44 ± 1	29 ± 1
4a	39 ± 1	22 ± 2	4b	7 ± 4	n.i.
5a	28 ± 3	n.i.	5b	9 ± 2	n.i.
6a	12 ± 3	n.i.	6b	37 ± 3	n.i.
7a	36 ± 2	n.i.	7b	13 ± 6	n.i.
8a	37 ± 3	n.i.	8b	24 ± 10	n.i.
9a	51 ± 1	7 ± 6	9b	50 ± 4	23 ± 1
10a	n.i.	n.i.	10b	n.i.	n.i.

a Purified linear peptides.

b *In situ* stapled peptides after 24 h incubation with 2,6-dicyanopyridine (DCP).

c %-Inhibition values at 10 μM and 1 μM compound concentrations; n.i., no inhibition.

### Detailed assessment of the stapling effect on ZiPro inhibition

**Fig. 3:**
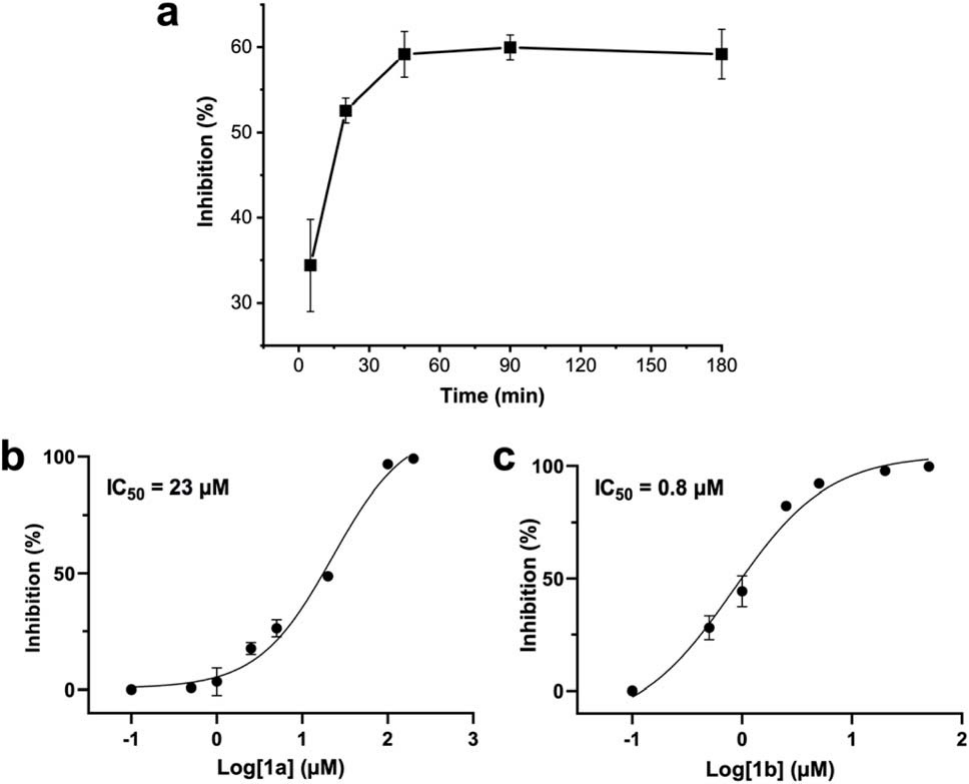
Zika virus NS2B-NS3 protease (ZiPro) inhibition of compounds 1a and 1b. (a) *In situ* generation of 1b during the ZiPro activity assay. A mixture of 1a and 2,6-dicyanopyridine (DCP) were incubated for 5 minutes prior addition to the ZiPro assay at a final concentration of 10 μM. Inhibition increase indicates *in situ* formation of 1b. (b) Dose–response curve and IC_50_ of 1a. (c) Dose–response curve and IC_50_ of 1b.

Addition of a mixture of 1a, DCP and TCEP to the ZiPro assay at a final peptide concentration of 10 μM resulted in a time-dependent increase of inhibition until plateauing after 90 minutes at 60% inhibition (Fig. 3a). This observation aligns with the screening results (Table 2) and clearly proves that the stapling reaction can proceed *in situ* in presence of the protease without any assay interference.

To further investigate whether the increased inhibition is related to conformational constraint, we synthesized 1b in larger scale by stapling crude 1a using the solution-phase approach. We established dose–response curves of purified 1a and 1b assuming Hill slopes of 1 (Fig. 3bc) and determined IC_50_ values of 23 μM and 0.8 μM for 1a and 1b, respectively. Thus, stapling of peptide 1a to 1b results in a more than 28 times increase of inhibition of ZiPro. As both compounds share the identical substrate recognition sequence (Lys-Arg-Lys), it can be reasoned that the increase in inhibition is directly related to conformational constraint triggered by DCP stapling. Notably, the results observed for the purified compounds align well with the initial screening results using *in situ* stapling, clearly indicating that the presented methodology is chemically robust and truly biocompatible.

### Structural aspects of peptide stapling

The virtue of peptide stapling is often narrowed down to the stabilisation of α-helical secondary structures, which is important for targeting protein–protein interactions and beyond.^3^ This study did not aim to stabilise helical structures and instead showcases the identification of constrained protease inhibitors. Most proteases recognise extended β-strands in their active site.^16^ Macrocyclization can pre-organise the protease inhibitor in the extended conformation and hence reduce the entropic penalty.^17^

**Fig. 4:**
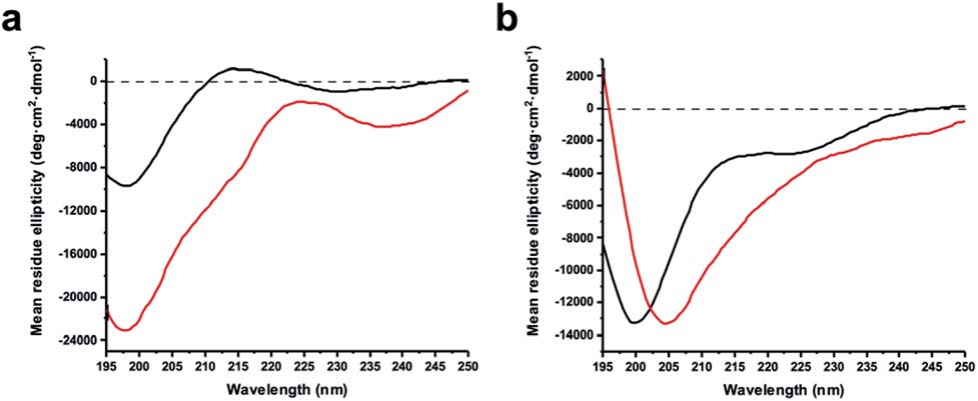
Circular dichroism spectra of linear and stapled peptides recorded at 0.1 mg ml^−1^ in 20 mM sodium phosphate pH 7.5, 1 mM TCEP. (a) 1a (black) and 1b (red). (b) aurein 1.2 analogues 18a (black) and 18b (red).

We recorded circular dichroism (CD) spectra of ZiPro inhibitors 1a and 1b (Fig. 4a) and analysed them with Beta Structure Selection (BeStSel) web server for the presence of common secondary structural elements like α-helix, β-sheet and turn (Table S2†).^18^ Both spectra indicate a mainly disordered structure, possibly allowing for an extended β-strand.^19^ This observation supports the idea that the increased inhibition of 1b compared to 1a is likely triggered by pre-organisation of the active conformation. It has been noted that particularly small macrocycles covering 3–4 amino acids, such as 1b, are privileged to form pre-organised protease inhibitors in the extended conformation.^17^

Although aurein 1.2 forms an amphipathic helix only in excess of trifluoroethanol,^13^ CD spectroscopy indicates that the *i*, *i* + 11 stapled analogue 18b may display up to 10% helicity in phosphate buffer compared to 0% for the linear analogue 18a (Fig. 4b, Table S2†). Future studies will further explore the scope of the presented stapling strategy for α-helical peptides. Given the four introduced pseudo-cysteine residues and the three potential stapling sites (*i*, *i* + 4; *i*, *i* + 7; *i*, *i* + 11), this will require intensive synthesis and structural evaluation.

## Conclusions

In summary, we developed a peptide-stapling strategy based on the staple 2,6-dicyanopyirdine and different pseudo-cysteine residues. The reaction proceeds in aqueous solution at physiological pH, requires no catalysts and is orthogonal to natural residues present in peptides and proteins.

We demonstrate that the stapling approach can be employed for *in situ* generation of constrained peptides directly in an enzymatic activity assay without any interferences. Even from a small series of only ten compounds, we could identify a stapled peptide that displayed much higher inhibition of an important viral drug target than its linear congener.

As we present a robust and straightforward synthetic method fully compatible with automated solid-phase synthesis, we are confident that the strategy will resonate well in drug discovery, chemical biology and beyond.

## Conflicts of interest

The authors declare no competing financial interests.

## Supplementary Material

SC-012-D0SC05125J-s001
