# Supplementary material for: A biocompatible stapling reaction for in situ generation of constrained peptides
Source: Chem Sci. 2020 Nov 4;12(2):669–74. doi: 10.1039/d0sc05125j (PMC8178976; doi:10.1039/d0sc05125j)
Supplement: SC-012-D0SC05125J-s001 [file SC-012-D0SC05125J-s001.pdf]

Electronic Supplementary Material (ESI) for Chemical Science.
This journal is © The Royal Society of Chemistry 2020

# SUPPORTING INFORMATION

# A biocompatible stapling reaction for *in situ* generation of constrained peptides

Richard Morewood and Christoph Nitsche

**List of abbreviations**

| | |
|---|---|
| ACN | Acetonitrile |
| CHAPS | 3-[(3-Cholamidopropyl)dimethylammonio]-1-propanesulfonate |
| Dab | L-2,4-diaminobutyric acid |
| Dap | L-2,3-diaminopropionic acid |
| DCC | *N*,*N'*-dicyclohexylcarbodiimide |
| DCM | Dichloromethane |
| DCP | 2,6-Dicyanopyridine |
| DIPEA | *N*,*N*-Diisopropylethylamine |
| DMF | Dimethylformamide |
| DMSO | Dimethyl sulfoxide |
| EDT | 1,2-Ethanedithiol |
| HBTU | 3-[Bis(dimethylamino)methyliumyl]-3*H*-benzotriazol-1-oxide hexafluorophosphate |
| HOBt | *N*-Hydroxybenzotriazole |
| TCEP | Tris(2-carboxyethyl)phosphine |
| TFA | Trifluoroacetic acid |
| TIPS | Triisopropylsilane |
| Tris | 2-Amino-2-(hydroxymethyl)-1,3-propanediol |

## Instrumentation and materials used for chemical synthesis

All NMR spectra were recorded on a Bruker Avance III 400 MHz equipped with a 5 mm Bruker probe head (PA BBO 400S1 BBF-H-D-05 Z SP). All measurements were performed at 25 °C. Spectra were processed using MestReNova. Chemical shifts for all NMR spectra are reported in parts per million (ppm) and were referenced by their residual solvent peaks. Coupling constants (*J*) are recorded in Hz and significant multiplicities are reported as singlet (s), doublet (d), triplet (t), multiplet (m), broad singlet (br s), and broad doublet (br d). Deuterated solvents were purchased from Cambridge Isotope Laboratories (USA). Low-resolution electrospray ionisation mass spectrometry analysis was performed on a Waters LCT Premier orthogonal acceleration time-of-flight mass spectrometer. High-resolution electrospray ionisation mass analysis was performed on a Thermo Scientific Orbitrap Elite mass spectrometer. Liquid chromatography-mass spectrometry analysis was performed on an Agilent HPLC/MS (1260/6120) equipped with a reverse-phase column (Poroshell 120 EC-C18 3.0 x 50 mm, 2.7 μm) held at 30 °C. A flow rate of 0.3 ml/min was utilised and elution was monitored by UV absorbance (254 and 200 nm). Acquisition and analysis were performed using LC/MSD Chemstation. Presentation of chromatograms was done using OriginPro. Analytical thin-layer chromatography analysis was performed on pre-coated silica gel aluminium-backed plates (Merck silica gel 60 $F_{254}$), using visualisation under UV light (254 nm). Synthesised amino acids were purified on a Biotage Isolera One automated flash chromatography system equipped with Biotage SNAP Ultra silica gel cartridges. Elution was monitored by UV absorbance (254 nm). Peptides were purified using preparative HPLC using a Waters 600 controller equipped with a reverse-phase column (SymmetryPrep C18, 100Å, 7 μm, 19 x 150 mm), autosampler (717 plus), diode array detector (2996), and a Waters Fraction Collector III. A flow rate of 10 ml/min was utilised and elution was monitored by UV absorbance (254 and 200 nm). Acquisition and analysis were performed using Waters Empower 2 software. Fmoc solid-phase peptide synthesis was performed manually using filtered polypropylene syringes purchased from Torviq (USA). Rink amide resin (capacity 0.67 mmol/g) was purchased from Auspep (Australia). Fmoc-protected amino acids were purchased from GL Biochem (China) and included Fmoc-Ala-OH, Fmoc-Arg(Pbf)-OH, Fmoc-Asn(Trt)-OH, Fmoc-Asp(OtBu)-OH, Fmoc-Cys(Trt)-OH, Fmoc-Gln(Trt)-OH, Fmoc-Glu(OtBu)-OH, Fmoc-Gly-OH, Fmoc-Ile-OH, Fmoc-Lys(Boc)-OH, Fmoc-Phe-OH, Fmoc-Ser(tBu)-OH, Fmoc-Thr(tBu)-OH, and Fmoc-Tyr(tBu)-OH. All other reagents were purchased from either AK Scientific (USA) or Sigma Aldrich (USA) and were used without further purification.

**Analytical LC-MS – method A**

This method used an isocratic eluent system (28% MeOH:water). TFA (0.1%) was used as an additive in both the MeOH and water. Each injection was monitored for 25 min.

**Analytical LC-MS – method B**

This method used a gradient eluent system of MeOH and water. After injection, the gradient started with 5% MeOH for 1 min, which was followed by a gradual MeOH increase to 90% over 10 min, before a final content of 90% MeOH was held until the end of the run. TFA (0.1%) was used as an additive in both the MeOH and water. The run was ended once all expected compounds eluted from the column.

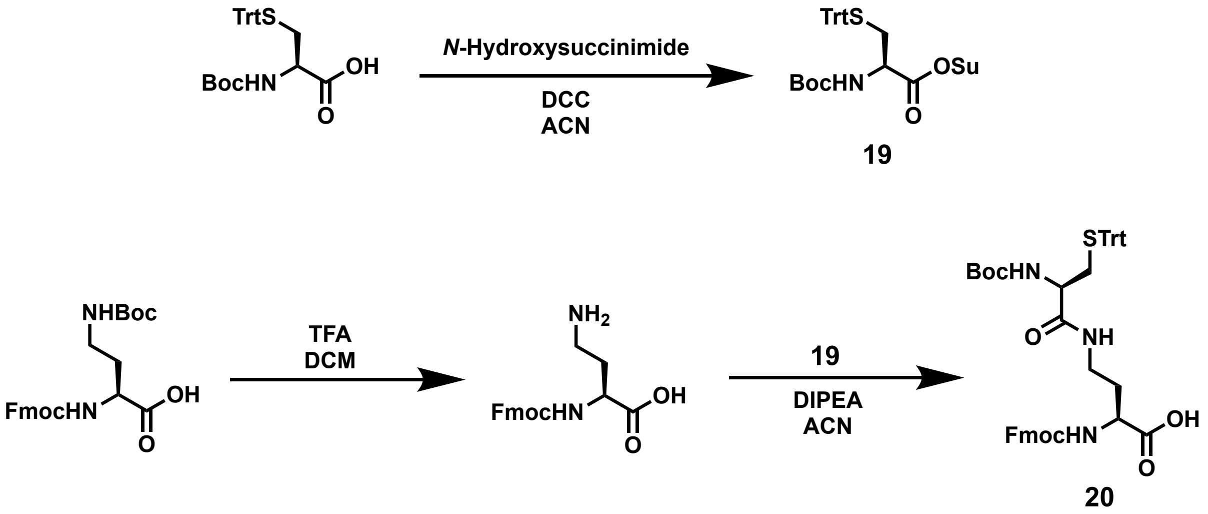


**Scheme S1.** Procedure for the synthesis of Fmoc-Dys(Boc,Trt)-OH (**20**).

### Synthesis of 2,5-dioxopyrrolidin-1-yl *N*-(*tert*-butoxycarbonyl)-*S*-trityl-L-cysteinate (19)

A 500 ml round bottom flask was charged with Boc-Cys(Trt)-OH (5.10 g, 11.0 mmol) and *N*-hydroxysuccinimide (1.39 g, 12.1 mmol) in ACN (200 ml). The mixture was cooled to 0 °C, DCC (2.72 g, 13.2 mmol) was added, and the mixture was stirred at 0 °C for 30 min, then room temperature overnight. The mixture was filtered, and the solvent was removed under reduced pressure. The crude product was purified using a 50 g silica gel cartridge (0 – 45% EtOAc:*n*-hexane) to yield **19** (5.85 g, 95%) as a white foam. **$^1$H NMR** (400 MHz, $CDCl_3$) $\delta$ 7.51 – 7.16 (m, 15H), 4.89 (d, $J$ = 8.0 Hz, 1H), 4.40 – 4.26 (m, 1H), 2.79 (s, 4H), 2.73 – 2.63 (m, 2H), 1.43 (s, 9H); **$^{13}$C NMR** (100 MHz, $CDCl_3$) $\delta$ 168.4, 166.9, 154.6, 144.2, 129.7, 128.3, 127.1, 80.8, 67.6, 51.3, 33.8, 28.4, 25.7; **LRMS** (ESI) *m/z*: 583.4 $[M+Na]^+$; **HRMS** (ESI) *m/z*: $[M+Na]^+$ Calcd for $C_{31}H_{32}N_2O_6S$ 583.1879, Found 583.1867; **TLC** $R_f$: 0.55 (50% EtOAc:*n*-hexane).

**Synthesis of (*S*)-2-((((9*H*-fluoren-9-yl)methoxy)carbonyl)amino)-4-((*R*)-2-((*tert*-butoxycarbonyl)amino)-3-(tritylthio)propanamido)butanoic acid (20)**

A 500 ml round bottom flask was charged with Fmoc-Dab(Boc)-OH (5.50 g, 12.5 mmol) and TFA (20 ml) in DCM (20 ml). The resulting mixture was stirred at room temperature for 1 h before being concentrated under reduced pressure. ACN (100 ml) was added and the mixture was cooled to 0 °C. DIPEA (7.83 ml, 44.9 mmol) and **19** (5.83 g, 10.4 mmol) were added and the mixture was stirred at 0 °C for 30 min, then room temperature overnight. The solvent was concentrated under reduced pressure and the residue was dissolved in EtOAc. The mixture was washed with an aqueous solution of 5% citric acid (x3), followed by brine, dried over $MgSO_4$, and filtered. The crude product was purified using a 50 g silica gel cartridge (0 – 50% EtOAc:*n*-hexane + 0.5% AcOH). The fractions containing the product were combined and evaporated under reduced pressure and the product was dissolved in DCM, washed with water (x3), followed by brine, dried over $MgSO_4$, and filtered. The solvent was removed under reduced pressure to yield **20** (6.01 g, 74%) as a white foam. **$^1$H NMR** (400 MHz, $CDCl_3$) $\delta$ 7.79 – 7.15 (m, 23H), 6.81 (br s, 1H), 5.86 (br d, $J$ = 6.6, 1H), 5.03 (br d, $J$ = 3.7, 1H), 4.44 – 4.28 (m, 3H), 4.20 (t, $J$ = 6.8, 1H), 3.92 (br s, 1H), 3.60 – 2.93 (m, 2H), 2.83 – 2.45 (m, 2H), 2.14 – 1.75 (m, 2H), 1.40 (s, 9H); **$^{13}$C NMR** (100 MHz, $CDCl_3$) $\delta$ 174.0, 156.4, 156.0, 144.4, 144.0, 143.7, 141.4, 141.4, 129.6, 128.2, 127.8, 127.2, 127.0, 125.3, 125.2, 120.1, 120.1, 81.1, 67.4, 67.2, 51.4, 47.3, 35.8, 33.9, 32.5, 28.4; **LRMS** *m/z*: 786.7 $[M+H]^+$; **HRMS** (ESI) *m/z*: $[M+H]^+$ Calcd for $C_{46}H_{47}N_3O_7S$ 786.3213, Found 786.3218; **TLC** $R_f$: 0.37 (10% MeOH:DCM).

**General procedure for solid-phase synthesis of 1a – 10a and 15a – 17a**

In a 10 ml filtered polypropylene syringe, Rink amide resin (Auspep 0.67 mmol/g) was swelled in DCM for 30 min. The resin was then washed with DMF (x3) before Fmoc deprotection with 25% piperidine in DMF for 10 min (x2). The resin was then washed with DMF (x3), DCM (x3), and DMF (x3). The resin was treated with a solution of *N*-Fmoc-protected amino acid (3 Eq), HBTU (3 Eq), HOBt (3 Eq), and DIPEA (4 Eq) in DMF (1.5 ml). The mixture was agitated for 1 h before being washed with DMF (x3), DCM (x3), and DMF (x3). The Fmoc deprotection, washing, and coupling steps were repeated until the desired peptide sequence has been formed. After a final Fmoc deprotection step, the resin was washed with DMF (x3), DCM (x3), diethyl ether (x3), and dried under reduced pressure for 2 h. The peptide was cleaved from the resin using a 1.5 ml mixture of TFA (91%), TIPS (3%), EDT (3%), and water (3%). The mixture was agitated for 2 h before being poured into ice-cold diethyl ether (40 ml). The precipitate was centrifuged and washed with cold diethyl ether (x3). The diethyl ether was decanted, and the crude peptide was dried under reduced pressure for 2 h. The product was purified by preparative HPLC using a gradient eluent system (5 – 90% MeOH:water + 0.1% TFA) over 20 min. The fractions containing the product were combined and lyophilised to yield the product. All purified peptides were stored at -20 °C. Identity was confirmed by high-resolution mass spectrometry (Table S1).

**General procedure for solid-phase synthesis of peptides 11a – 14a and 18a**

The resin was prepared according to the general procedure and either Fmoc-Dap(Boc)-OH (**11a**), Fmoc-Dab(Boc)-OH (**12a**), Fmoc-Orn(Boc)-OH (**13a**) or Fmoc-Lys(Boc)-OH (**14a** and **18a**) was coupled to it. After washing with DMF (x3) and DCM (x3), the resin was treated with a solution of 25% TFA and 75% DCM twice for three minutes each. After another wash with DCM (x3) and DMF (x3), the resin was agitated for 1 h with a solution containing Boc-Cys(Trt)-OH (3 Eq), HBTU (3 Eq), HOBt (3 Eq), and DIPEA (5 Eq) in DMF. Preparation of the peptide continued according to the general procedure.

**General procedure for synthesis and determination of yield of 1b – 18b in solution**

20 μl of DCP (20 mM in MeOH), followed by 20 μl of **1a** – **16a** and **18a** (10 mM in water) were added to 300 μl of buffer (10 mM Tris pH 7.5, 4 mM TCEP). Due to precipitation of **17b** in aqueous buffer, **17a** was cyclised in methanolic buffer (75% MeOH, 10 mM Tris pH 7.5, 4 mM TCEP). The solutions were incubated for either 24 h (**1a** – **10a** and **15a** – **18a**) or 6 h (**11a** – **14a**) before analysis by LC-MS (method B). Yields were determined by integrating peaks (excluding DCP) of the 254 nm chromatogram. Identity was confirmed by high-resolution mass spectrometry (Table S1).

**Synthesis and isolation of 1b in solution**

1 ml of DCP (20 mM in MeOH), followed by 1 ml of **1a** (10 mM in water) were added to 15 ml of buffer (10 mM Tris pH 7.5, 4 mM TCEP). The mixture was incubated for 24 h and reaction completion was confirmed by LC-MS (method B). The mixture was lyophilised, and the stapled peptide was purified by preparative HPLC using a gradient eluent system (5 – 90% MeOH:water + 0.1% formic acid) over 20 min. Identity was confirmed by high-resolution mass spectrometry (Table S1).

**Synthesis and isolation of 1d**

Crude **1b** was incubated in a 2% solution of TFA in water for 1 h. After the formation of **1d** was confirmed by LC-MS (method B), the product was purified by preparative HPLC using a gradient eluent system (5 – 90% MeOH:water + 0.1% TFA) over 20 min. Identity was confirmed by high-resolution mass spectrometry (Table S1).

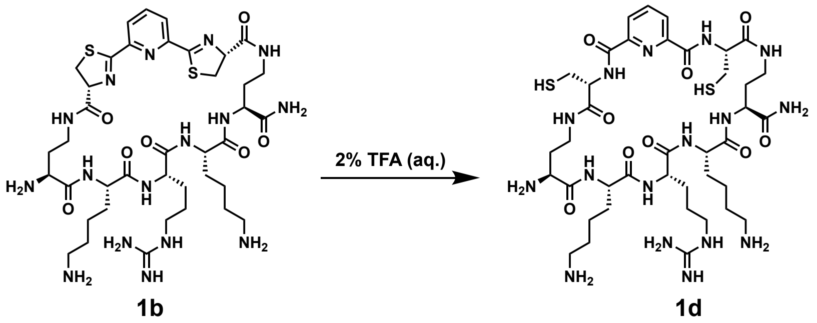


**Scheme S2.** Hydrolysis of **1b** to **1d** in 2% TFA in water (full conversion after 1 h).

**On-resin synthesis of 1b and LC-MS based assay to determine optimal DCP equivalents**

Four samples of **1a** were synthesised on-resin (Merck Millipore Rink Amide MBHA LL 0.38 mmol/g) according to the general procedure. The last Fmoc group was removed and the resin was washed with DMF (x3) and DCM (x3). The resin was treated with a solution of 25% TFA, 5% TIPS, 5% EDT, and 65% DCM twice for three minutes each. The resin was washed with DCM (x3) and DMF (x3). Each sample was agitated with a DMF solution containing either 0.5, 1.0, 1.5, or 2.0 Eq of DCP and 9 Eq of DIPEA for 2 h. The resin was washed with DMF (x3) and preparation continued according to the general procedure. The four samples of crude **1b** were then measured by LC-MS (method B) and the ions of **1a** [*m*/*z* = 419, 836 (linear peptide); 418, 834 (cyclic disulfide)], **1b** [*m*/*z* = 466, 931], and **1c** [*m*/*z* = 531, 1060] were extracted using Lablicate OpenChrom to produce extracted ion chromatograms (EIC). The EIC of the four samples were integrated to give ratios of **1a**, **1b**, and **1c**.

**Preparation of external standard calibration curve of 1b**

Standard solutions of purified **1b** were prepared at six concentrations (1000, 333, 111, 37, 12, and 4 µM in water). 10 µl of each standard was injected into the LC-MS (method A). Using the peak area (254 nm) of each **1b** standard, a calibration curve was prepared by applying linear regression.

**LC-MS based assay to determine optimal equivalents of DCP (Figure 1a)**

Five samples were prepared separately by adding 10 µl of DCP (5, 10, 20, 40, and 80 mM in MeOH), followed by 10 µl of the purified linear peptide (10 mM **1a** in water) to 150 µl of buffer (10 mM Tris pH 7.5, 1 mM TCEP). After 1 h, 10 µl of the mixture was injected into the LC-MS (method A). Using the peak area (254 nm) of **1b** in each mixture, the concentration was interpolated from the calibration curve and used to calculate the yield.

**LC-MS based assay to determine stapling kinetics (Figure 1b)**

Samples were prepared by adding 10 µl of DCP (20 mM in MeOH), followed by 10 µl of the purified linear peptide (10 mM **1a** in water) to 150 µl of buffer (10 mM Tris pH 7.5, 1 mM TCEP). 10 µl were injected into the LC-MS (method A) after 10, 20, 30, 40, 50, and 80 minutes. Using the peak area (254 nm) of **1b** in each mixture, the concentration was interpolated from the calibration curve and used to calculate the yield.

**LC-MS based assay to determine optimal equivalents of TCEP (Figure 1c)**

Four samples were prepared separately by adding 10 µl of DCP (20 mM in MeOH), followed by 10 µl of the purified linear peptide (10 mM **1a** in water) to one of four 150 µl of buffer solutions (10 mM Tris pH 7.5) containing different concentrations of TCEP (0.5, 1, 2, and 4 mM). After 1 h, 10 µl of the mixture was injected into the LC-MS (method A). Using the peak area (254 nm) of **1b** in each mixture, the concentration was interpolated from the calibration curve and used to calculate the yield.

**LC-MS based assay to analyse the effect of peptide concentration (Figure 1d)**

Three samples were prepared separately. The first sample (588 µM of **1a**) was prepared by adding 10 µl of DCP (20 mM in MeOH), followed by 10 µl of the purified linear peptide (10 mM **1a** in water) to 150 µl of buffer (10 mM Tris pH 7.5, 4 mM TCEP). The second sample (118 µM of **1a**) was prepared by adding 10 µl of DCP (4 mM in MeOH), followed by 10 µl of the purified linear peptide (2 mM **1a** in water) to 150 µl of buffer (2 mM Tris pH 7.5, 0.8 mM TCEP). The third sample (59 µM of **1a**) was prepared by adding 10 µl of DCP (2 mM in MeOH), followed by 10 µl of the purified linear peptide (1 mM **1a** in water) to 150 µl of buffer (1 mM Tris pH 7.5, 0.4 mM TCEP). After 1 h, 10 µl of the mixture was injected into the LC-MS (method A). Using the peak area (254 nm) of **1b** in each mixture, the concentration was interpolated from the calibration curve and used to calculate the yield.

**Expression and purification of Zika virus protease NS2B-NS3 (ZiPro)**

The unlinked Zika virus protease NS2B-NS3 construct referred to as bZiPro was obtained from Addgene (plasmid #86846) and expressed and purified exactly as described previously.[1]

**Preparation of samples for ZiPro assay**

Linear peptide samples **1a – 10a** were prepared by adding 2 µl of **1a – 10a** (10 mM in water) to 198 µl of buffer (10 mM Tris pH 7.5, 1 mM TCEP). The presence of TCEP is important to reduce cyclic disulfides. Stapled peptides **1b – 10b** were prepared *in situ* by adding 2.5 µl of DCP (10 mM in DMSO) and 2 µl of **1a – 10a** (10 mM in water) to 195.5 µl of buffer (10 mM Tris pH 7.5, 1 mM TCEP). Samples were incubated overnight, and the presence of stapled peptides **1b – 10b** was confirmed by LC-MS (method B).

**ZiPro inhibition assay (Table 2)**

The assay was conducted in 96-well plates (black U-bottom, Greiner Bio-One) in a total volume of 100 µl using 10 mM Tris pH 8.5, 1 mM CHAPS, 20% glycerol. All measurements were performed in triplicate. Purified peptides **1a – 10a** and crude mixtures containing peptides **1b – 10b** (prepared as described above) were incubated with ZiPro (0.3 nM) at final concentrations of 1 and 10 µM. The enzymatic reaction was initiated by addition of the substrate Bz-Nle-Lys-Lys-Arg-AMC (Biosyntan) at a final concentration of 5 µM. Fluorescence was monitored by a fluorophotometer (Spectramax M2e plate reader, Molecular Devices) for 5 min at 460 nm using 360 nm excitation. Initial velocities were derived from the linear range, and 100% enzyme activity was defined as the initial velocity of blank wells containing no peptide. Percent inhibition was calculated as a fraction of 100% enzyme activity. The concentrations of DCP used were shown to have no significant effect on ZiPro activity.

***In situ* stapling of 1b during ZiPro inhibition assay (Figure 3a)**

All measurements were performed in triplicate. 2 µl of DCP (20 mM in DMSO) and 2 µl of **1a** (10 mM in water) were mixed in 196 µl buffer (10 mM Tris pH 7.5, 4 mM TCEP). A blank was prepared by adding 2 µl of DCP (20 mM in DMSO) to 198 µl buffer (10 mM Tris pH 7.5, 4 mM TCEP). After 5 minutes, 10 µl of the reaction and blank mixtures were added to ZiPro (0.3 nM) in assay buffer (10 mM Tris pH 8.5, 1 mM CHAPS, 20% glycerol) to a total volume of 100 µl and incubated at room temperature. After incubation over 5, 20, 45, 90 and 180 minutes, substrate (5 µM) was added, and the enzyme activity was monitored as described above. Percent inhibition was calculated as a fraction of 100% enzyme activity considering each individual set of blanks.

**Determination of $IC_{50}$ values of 1a and 1b (Figure 3b-c)**

All measurements were performed in triplicate using the ZiPro assay described above. Stock solutions (1 mM and 2 mM) of **1a** were prepared in buffer (10 mM Tris pH 7.5, 4 mM TCEP) and used immediately. ZiPro inhibition of **1a** was measured at 100, 50, 20, 5, 2.5, 1, and 0.5 µM. Stock solutions of purified **1b** were made in water. ZiPro inhibition of **1b** was measured at 50, 20, 5, 2.5, 1, 0.5, and 0.1 µM. The $IC_{50}$ values were calculated using GraphPad Prism 8 using a 3-parameter fit.

**Circular dichroism (CD) spectroscopy**

All CD spectra were obtained on an Applied Photophysics Chirascan CD spectrometer at 24 °C using a 1 mm path length quartz cell. Measurements were taken over 195 – 250 nm (0.5 nm steps). Each sample was measured in buffer (20 mM sodium phosphate pH 7.5, 1 mM TCEP) at a concentration of 0.1 mg/ml. Spectra were presented in OriginPro and smoothed using a Savitzky-Golay filter (points = 12, polynomial order = 3). CD spectra of **1a**, **1b** and **18a** were obtained from purified samples. The CD spectrum of **18b** was obtained by cyclising purified **18a** in buffer (20 mM sodium phosphate pH 7.5, 1 mM TCEP) at a concentration of 0.1 mg/ml with 1 Eq of DCP (MeOH cosolvent). After 24 h, full conversion to **18b** and high purity was confirmed by LC-MS and the CD measurement was conducted. The secondary structural elements of **1a**, **1b**, **18a**, and **18b** were estimated from the 200 – 250 nm values using BeStSel web server and are presented in Table S2.[2]

**Table S1.** High-resolution mass spectrometry (HRMS) results for all analysed peptides.

| Peptide | Molecular formula | HRMS calculated | HRMS found |
|---|---|---|---|
| **1a**[a] | $C_{32}H_{63}N_{15}O_7S_2$ | 834.4555 $[M+H]^+$ | 834.4542 $[M+H]^+$ |
| **1b** | $C_{39}H_{62}N_{16}O_7S_2$ | 931.4507 $[M+H]^+$ | 931.4501 $[M+H]^+$ |
| **1d** | $C_{39}H_{66}N_{16}O_9S_2$ | 967.4718 $[M+H]^+$ | 967.4702 $[M+H]^+$ |
| **2a**[a] | $C_{25}H_{45}N_{13}O_8S_2$ | 720.3034 $[M+H]^+$ | 720.3029 $[M+H]^+$ |
| **2b** | $C_{32}H_{44}N_{14}O_8S_2$ | 817.2986 $[M+H]^+$ | 817.3002 $[M+H]^+$ |
| **3a**[a] | $C_{47}H_{83}N_{17}O_9S_2$ | 1094.6079 $[M+H]^+$ | 1094.6064 $[M+H]^+$ |
| **3b** | $C_{54}H_{82}N_{18}O_9S_2$ | 1191.6032 $[M+H]^+$ | 1191.6011 $[M+H]^+$ |
| **4a**[a] | $C_{62}H_{93}N_{17}O_{11}S_2$ | 1316.6760 $[M+H]^+$ | 1316.6755 $[M+H]^+$ |
| **4b** | $C_{69}H_{92}N_{18}O_{11}S_2$ | 1413.6713 $[M+H]^+$ | 1413.6691 $[M+H]^+$ |
| **5a**[a] | $C_{32}H_{63}N_{15}O_7S_2$ | 834.4555 $[M+H]^+$ | 834.4547 $[M+H]^+$ |
| **5b** | $C_{39}H_{62}N_{16}O_7S_2$ | 931.4507 $[M+H]^+$ | 931.4507 $[M+H]^+$ |
| **6a**[a] | $C_{29}H_{56}N_{14}O_8S_2$ | 793.3925 $[M+H]^+$ | 793.3907 $[M+H]^+$ |
| **6b** | $C_{36}H_{55}N_{15}O_8S_2$ | 890.3878 $[M+H]^+$ | 890.3876 $[M+H]^+$ |
| **7a**[a] | $C_{38}H_{75}N_{19}O_8S_2$ | 990.5566 $[M+H]^+$ | 990.5549 $[M+H]^+$ |
| **7b** | $C_{45}H_{74}N_{20}O_8S_2$ | 1087.5518 $[M+H]^+$ | 1087.5499 $[M+H]^+$ |
| **8a**[a] | $C_{43}H_{83}N_{19}O_{11}S_2$ | 1106.6039 $[M+H]^+$ | 1106.6008 $[M+H]^+$ |
| **8b** | $C_{50}H_{82}N_{20}O_{11}S_2$ | 1203.5992 $[M+H]^+$ | 1203.5974 $[M+H]^+$ |
| **9a**[a] | $C_{44}H_{87}N_{21}O_9S_2$ | 1118.6515 $[M+H]^+$ | 1118.6487 $[M+H]^+$ |
| **9b** | $C_{51}H_{86}N_{22}O_9S_2$ | 608.3273 $[M+2H]^{2+}$ | 608.3260 $[M+H]^{2+}$ |
| **10a**[a] | $C_{29}H_{56}N_{12}O_7S_3$ | 781.3635 $[M+H]^+$ | 781.3631 $[M+H]^+$ |
| **10b** | $C_{36}H_{55}N_{13}O_7S_3$ | 878.3588 $[M+H]^+$ | 878.3588 $[M+H]^+$ |
| **11a** | $C_{27}H_{55}N_{13}O_6S_2$ | 722.3918 $[M+H]^+$ | 722.3918 $[M+H]^+$ |
| **11b** | $C_{34}H_{52}N_{14}O_6S_2$ | 817.3714 $[M+H]^+$ | 817.3701 $[M+H]^+$ |
| **12a** | $C_{28}H_{57}N_{13}O_6S_2$ | 736.4074 $[M+H]^+$ | 736.4075 $[M+H]^+$ |
| **12b** | $C_{35}H_{54}N_{14}O_6S_2$ | 831.3870 $[M+H]^+$ | 831.3850 $[M+H]^+$ |
| **13a** | $C_{29}H_{59}N_{13}O_6S_2$ | 750.4231 $[M+H]^+$ | 750.4236 $[M+H]^+$ |
| **13b** | $C_{36}H_{56}N_{14}O_6S_2$ | 845.4027 $[M+H]^+$ | 845.4019 $[M+H]^+$ |
| **14a**[a] | $C_{30}H_{59}N_{13}O_6S_2$ | 762.4231 $[M+H]^+$ | 762.4230 $[M+H]^+$ |
| **14b** | $C_{37}H_{58}N_{14}O_6S_2$ | 859.4183 $[M+H]^+$ | 859.4186 $[M+H]^+$ |
| **15a** | $C_{28}H_{46}N_{10}O_{14}S_2$ | 811.2715 $[M+H]^+$ | 811.2728 $[M+H]^+$ |
| **15b** | $C_{35}H_{43}N_{11}O_{14}S_2$ | 906.2511 $[M+H]^+$ | 906.2510 $[M+H]^+$ |
| **16a** | $C_{25}H_{45}N_{11}O_9S_2$ | 708.2921 $[M+H]^+$ | 708.2921 $[M+H]^+$ |
| **16b** | $C_{32}H_{42}N_{12}O_9S_2$ | 803.2717 $[M+H]^+$ | 803.2714 $[M+H]^+$ |
| **17a** | $C_{45}H_{70}N_{12}O_{11}S_2$ | 1019.4807 $[M+H]^+$ | 1019.4805 $[M+H]^+$ |
| **17b** | $C_{52}H_{67}N_{13}O_{11}S_2$ | 1114.4603 $[M+H]^+$ | 1114.4603 $[M+H]^+$ |
| **18a** | $C_{72}H_{124}N_{20}O_{20}S_2$ | 1653.8820 $[M+H]^+$ | 1653.8817 $[M+H]^+$ |
| **18b** | $C_{79}H_{121}N_{21}O_{20}S_2$ | 1748.8617 $[M+H]^+$ | 1748.8617 $[M+H]^+$ |

[a] HRMS results of the cyclic disulfide are reported.

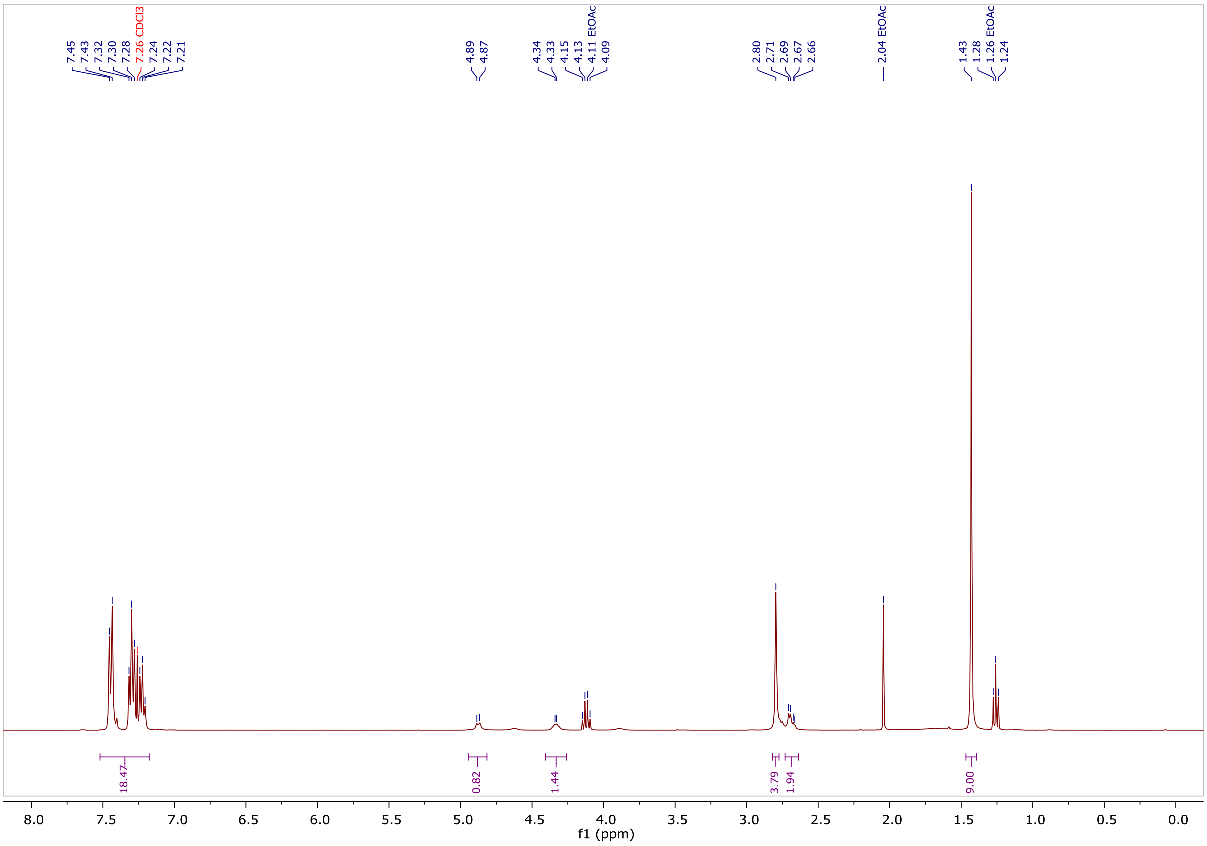


**Figure S1.** $^{1}$H-NMR spectrum (400 MHz, $CDCl_3$) of compound **19**.

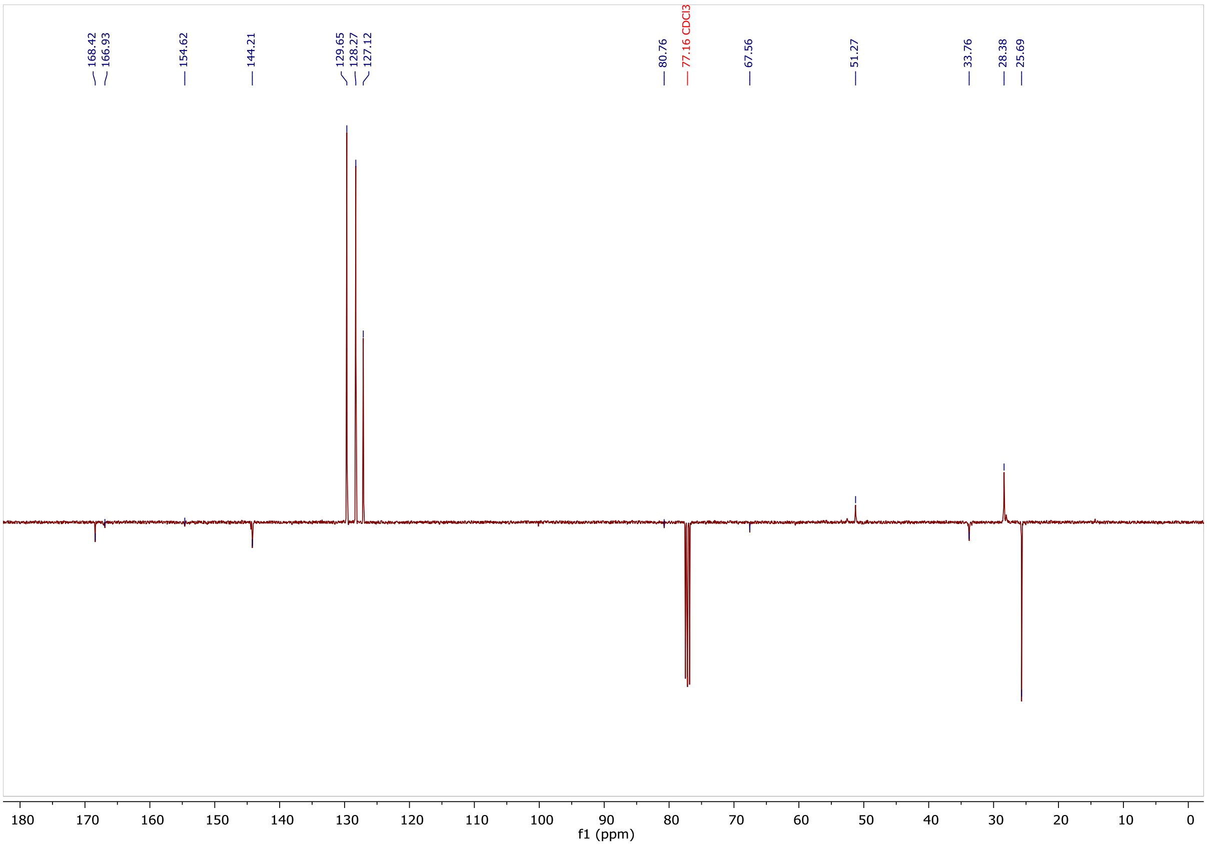


**Figure S2.** $^{13}$C-NMR APT spectrum (100 MHz, $CDCl_3$) of compound **19**.

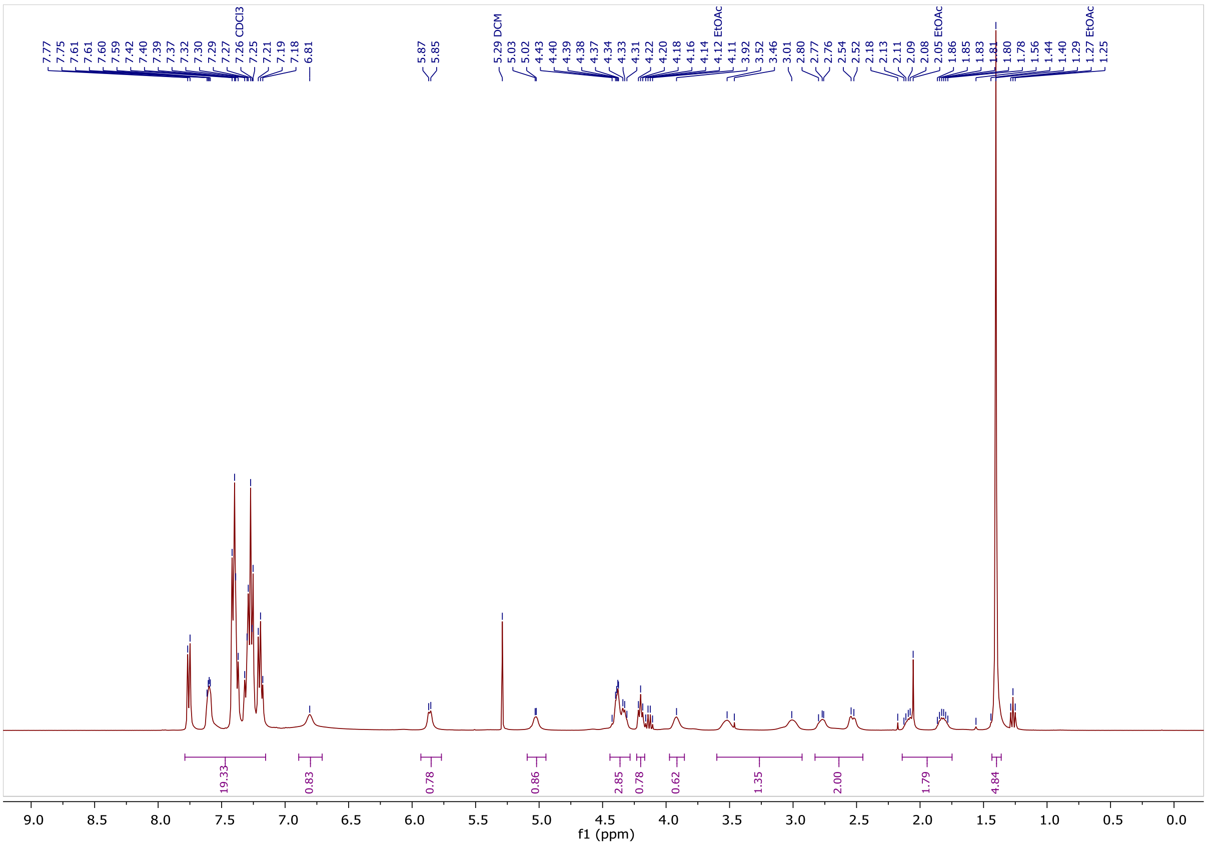

**Figure S3.** $^{1}$H-NMR spectrum (400 MHz, $CDCl_3$) of Fmoc-Dys(Trt,Boc)-OH (**20**).

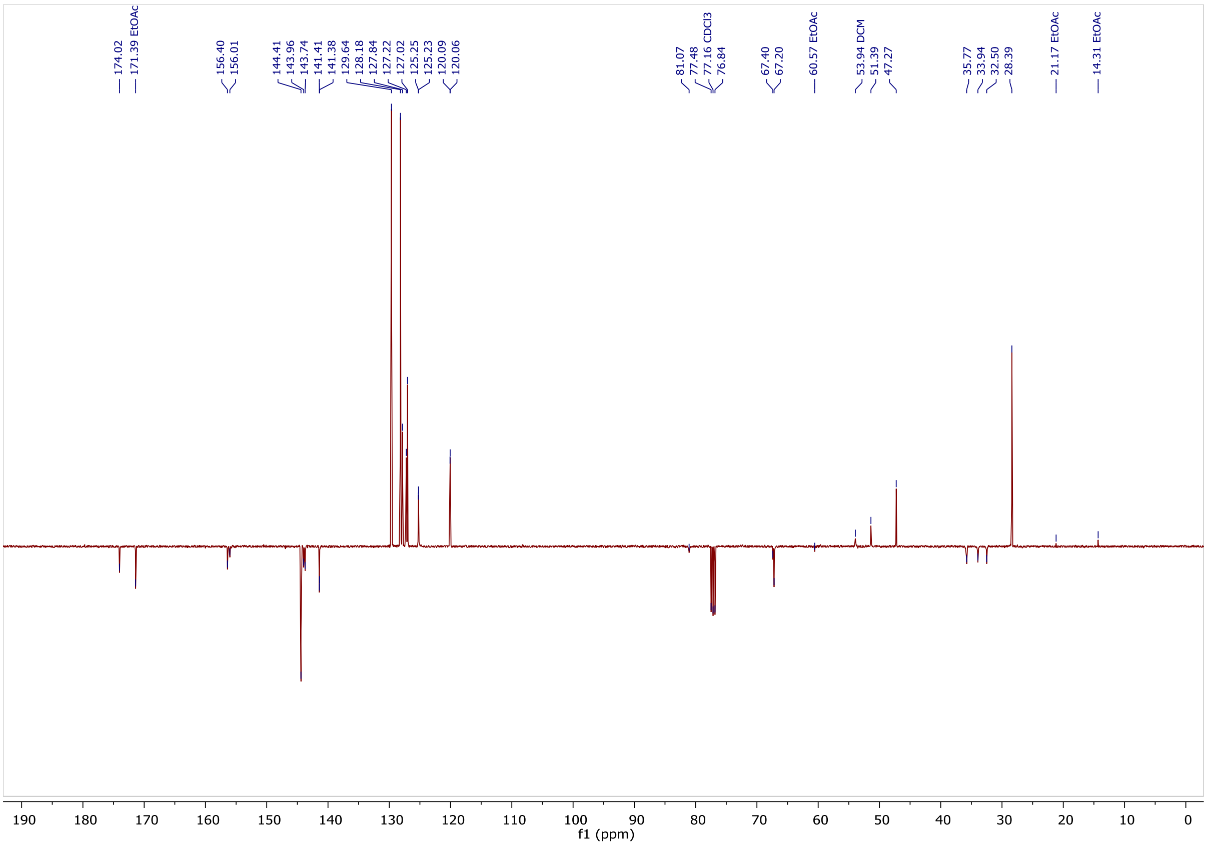


**Figure S4.** $^{13}$C-NMR APT spectrum (100 MHz, $CDCl_3$) of Fmoc-Dys(Trt,Boc)-OH (**20**).

## Chromatograms of all linear and cyclic peptides

All chromatograms reported below were acquired using LC-MS method B. Compounds were identified by their corresponding *m*/*z* ratio and are labelled as linear peptides (**a**), stapled peptides (**b**), and double DCP-capped by-products (**c**).

## Analytical chromatograms of purified peptides 1a – 18a

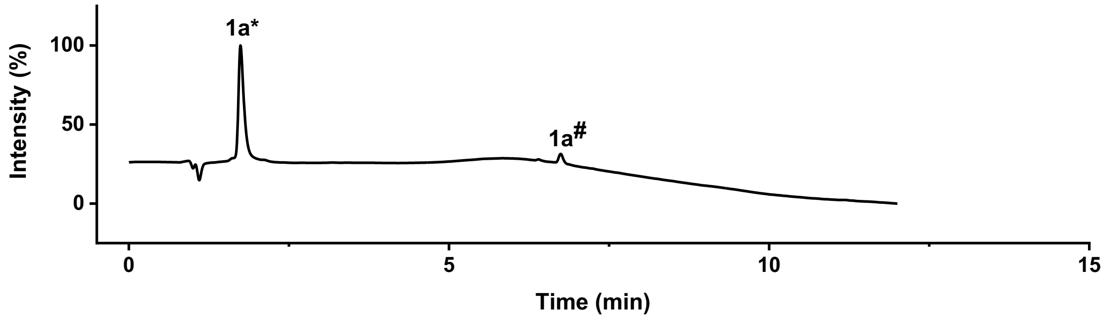


**Figure S5.** 200 nm chromatogram of purified **1a** showing oxidised cyclic disulfide* (1.74 min) and oxidised cyclic disulfide dimer# (6.74 min).

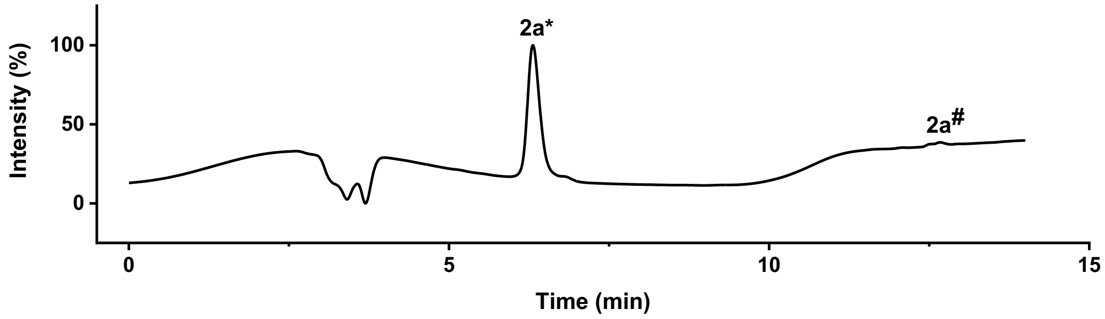


**Figure S6.** 200 nm chromatogram of purified **2a** showing oxidised cyclic disulfide* (6.31 min) and oxidised cyclic disulfide dimer# (12.68 min).

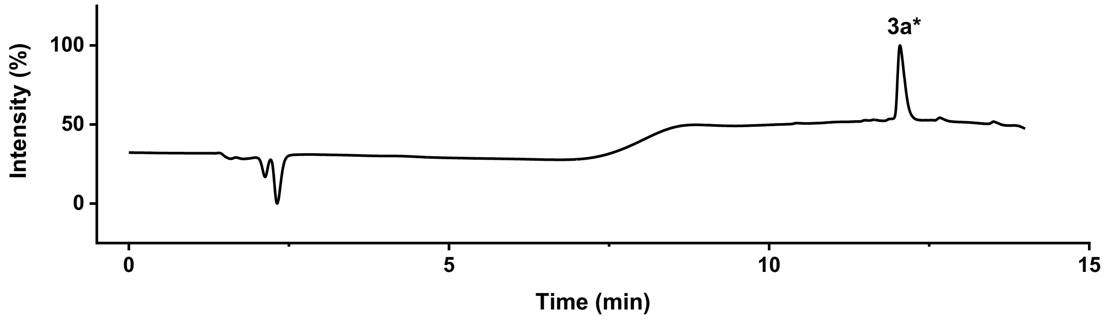


**Figure S7.** 200 nm chromatogram of purified **3a** showing oxidised cyclic disulfide* (12.04 min).

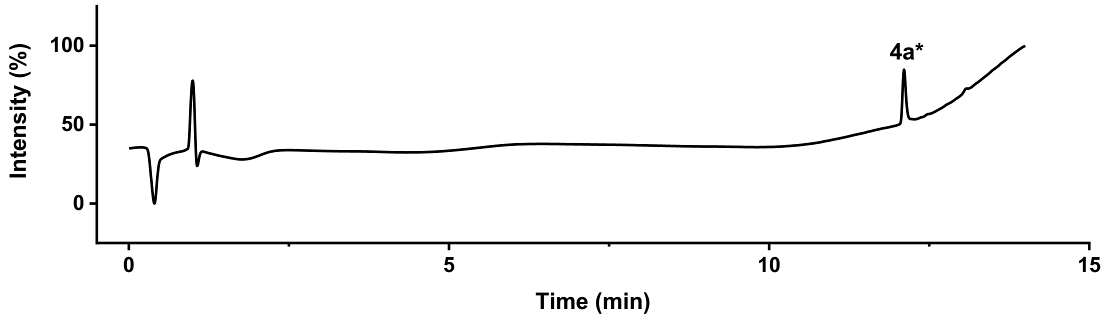


**Figure S8.** 200 nm chromatogram of purified **4a** showing oxidised cyclic disulfide* (12.10 min).

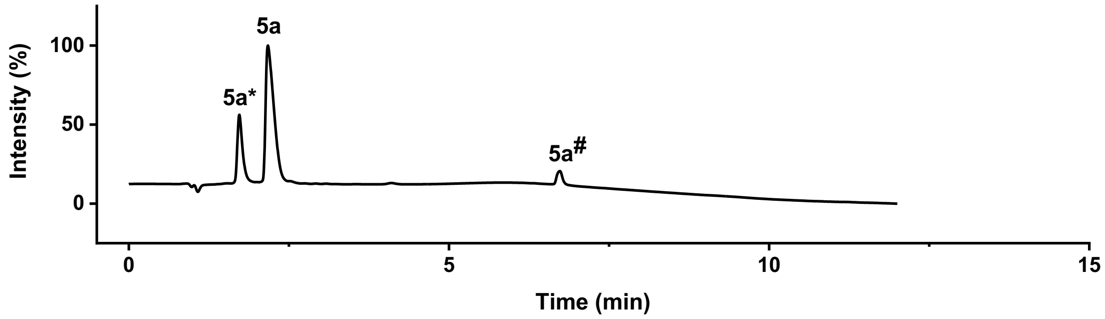


**Figure S9.** 200 nm chromatogram of purified **5a** showing oxidised cyclic disulfide* (1.72 min), linear peptide (2.17 min), and oxidised cyclic disulfide dimer# (6.73 min).

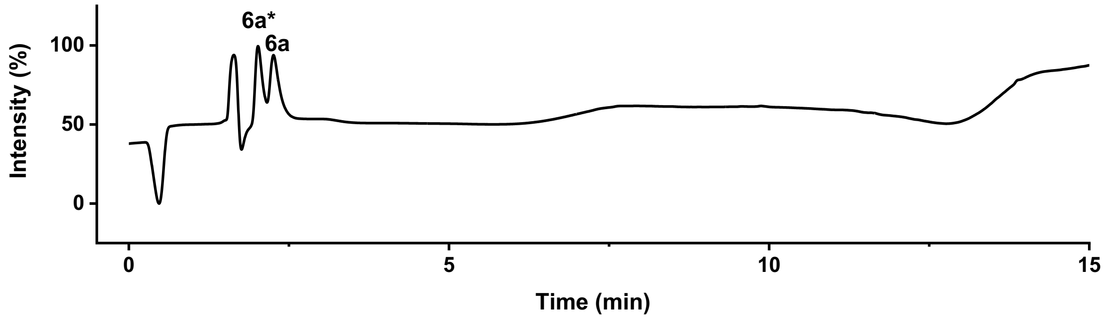


**Figure S10.** 200 nm chromatogram of purified **6a** showing oxidised cyclic disulfide* (2.02 min) and linear peptide (2.26 min).

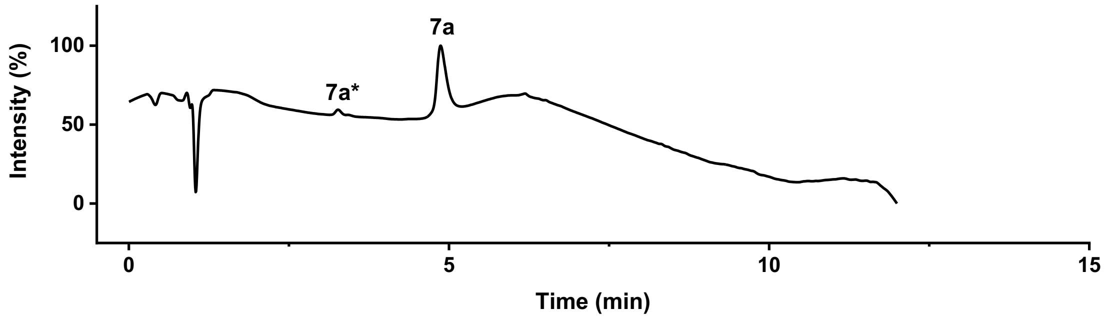


**Figure S11.** 200 nm chromatogram of purified **7a** showing oxidised cyclic disulfide* (3.26 min) and linear peptide (4.87 min).

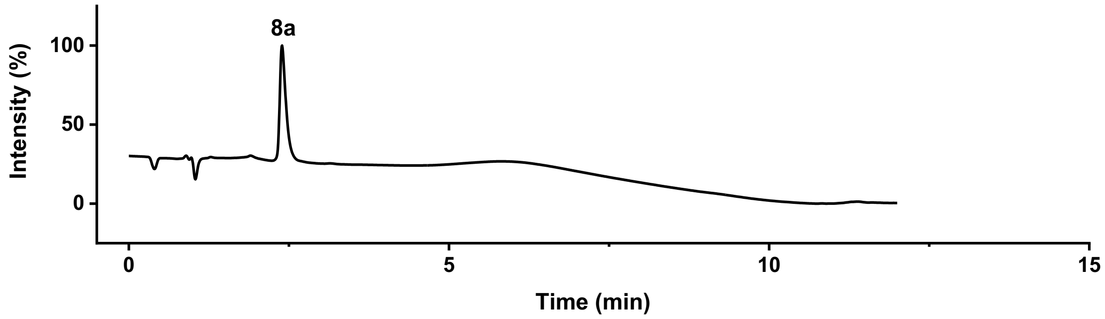


**Figure S12.** 200 nm chromatogram of purified **8a** showing linear peptide (2.39 min).

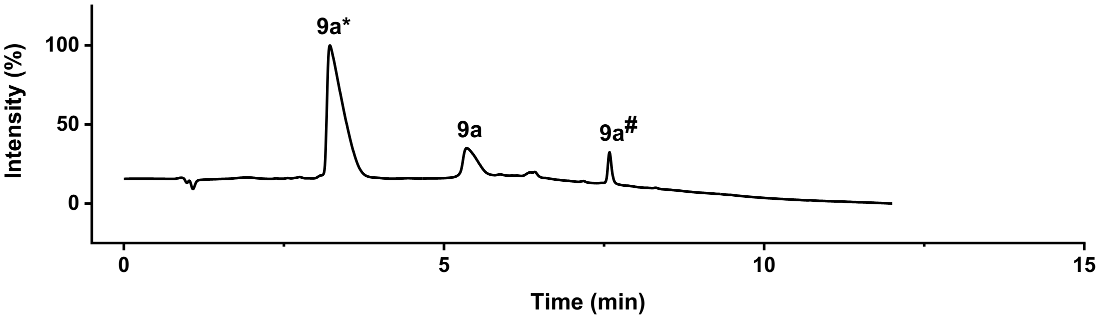


**Figure S13.** 200 nm chromatogram of purified **9a** showing oxidised cyclic disulfide* (3.21 min), linear peptide (5.36 min), and oxidised cyclic disulfide dimer# (7.58 min).

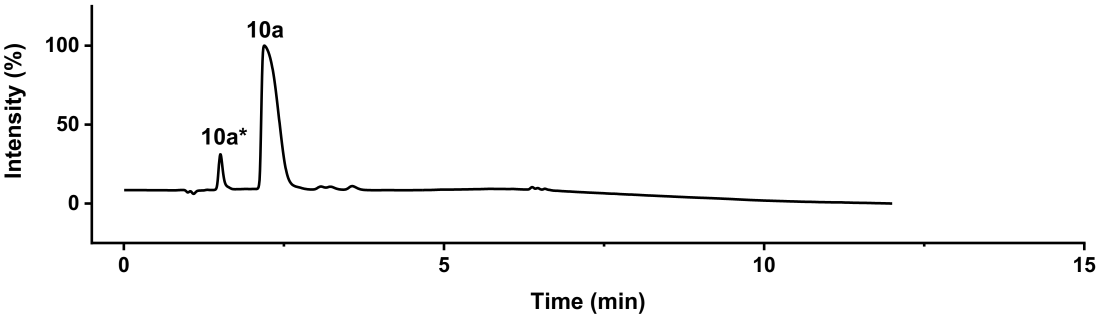


**Figure S14.** 200 nm chromatogram of purified **10a** showing oxidised cyclic disulfide* (1.51 min) and linear peptide (2.19 min).

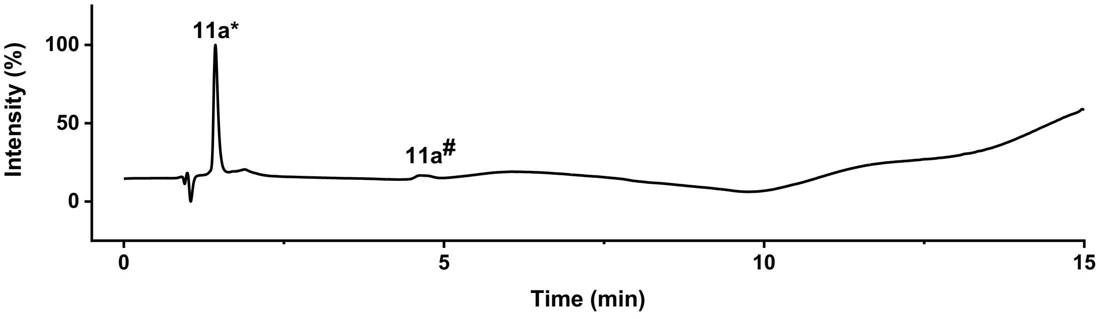


**Figure S15.** 200 nm chromatogram of purified **11a** showing oxidised cyclic disulfide* (1.43 min) and oxidised cyclic disulfide dimer# (4.61 min).

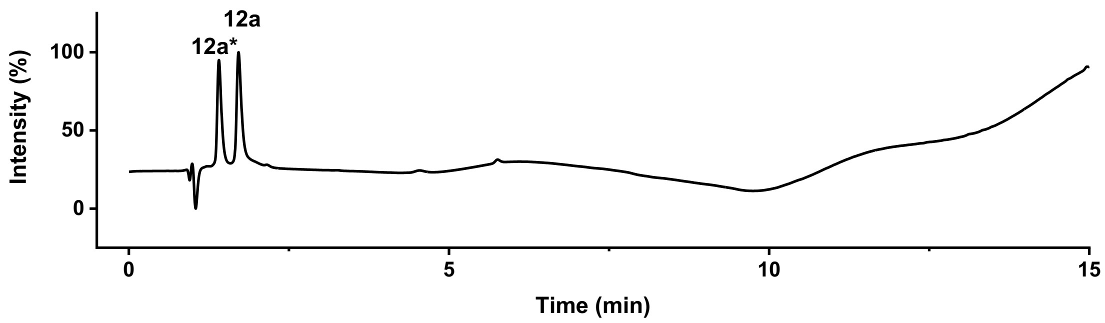


**Figure S16.** 200 nm chromatogram of purified **12a** showing oxidised cyclic disulfide* (1.41 min) and linear peptide (1.71 min).

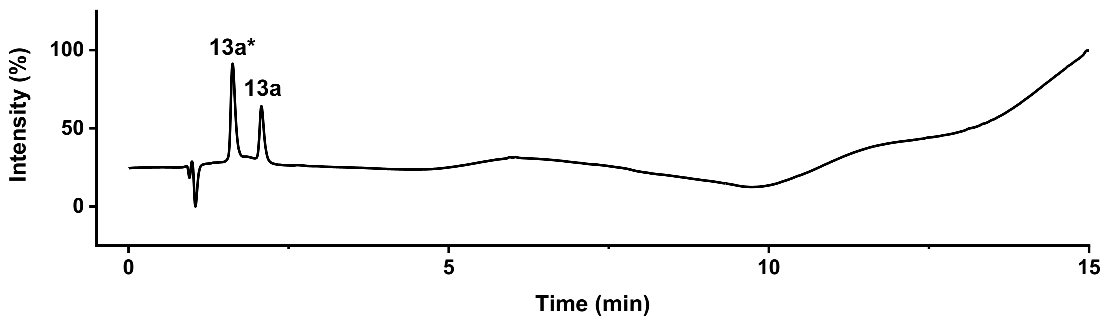


**Figure S17.** 200 nm chromatogram of purified **13a** showing oxidised cyclic disulfide* (1.63 min) and linear peptide (2.07 min).

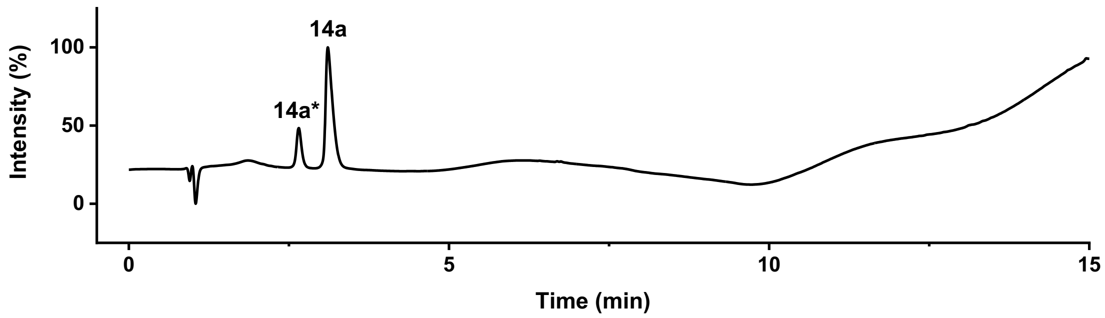


**Figure S18.** 200 nm chromatogram of purified **14a** showing oxidised cyclic disulfide* (2.65 min) and linear peptide (3.10 min).

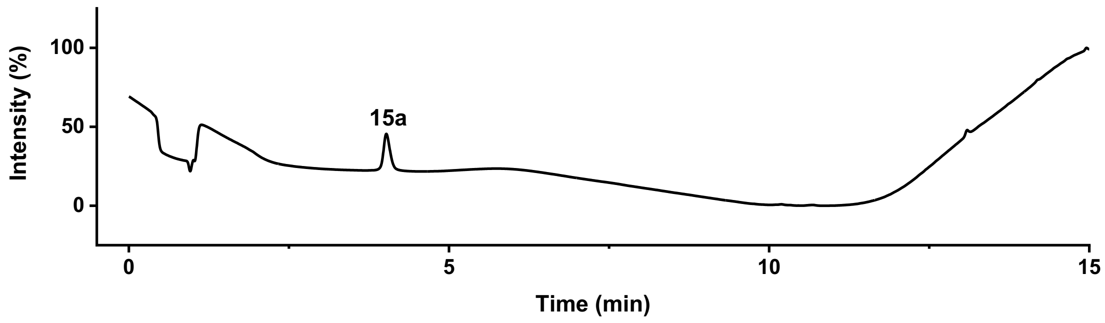


**Figure S19.** 200 nm chromatogram of purified **15a** showing linear peptide (4.02 min).

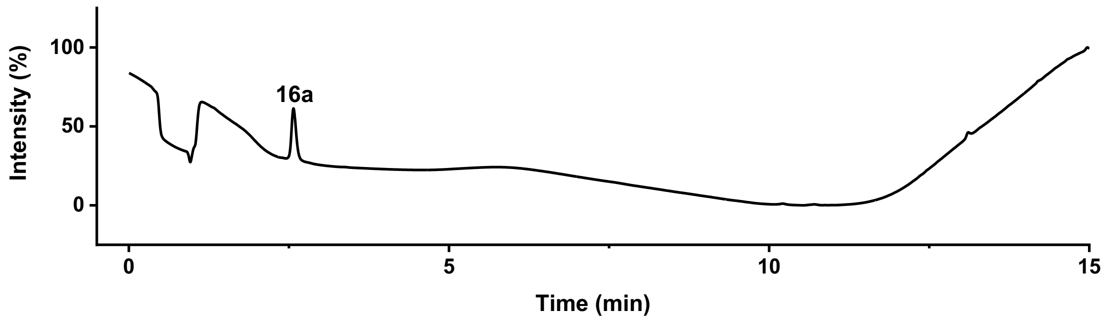


**Figure S20.** 200 nm chromatogram of purified **16a** showing linear peptide (2.57 min).

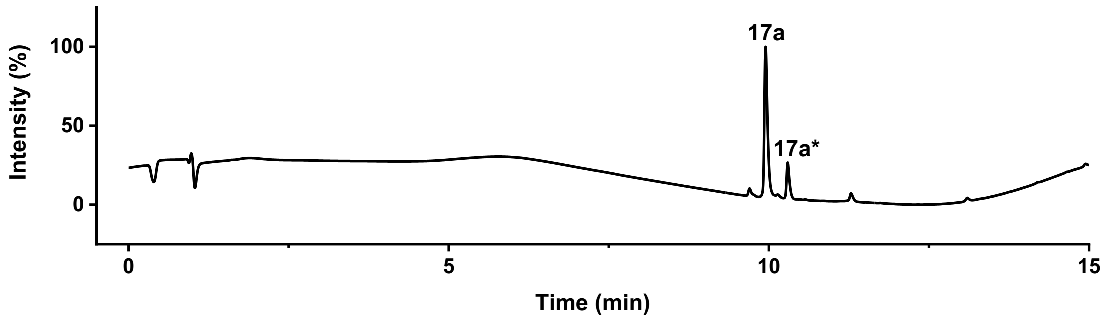


**Figure S21.** 200 nm chromatogram of purified **17a** showing linear peptide (9.95 min) and oxidised cyclic disulfide* (10.30 min).

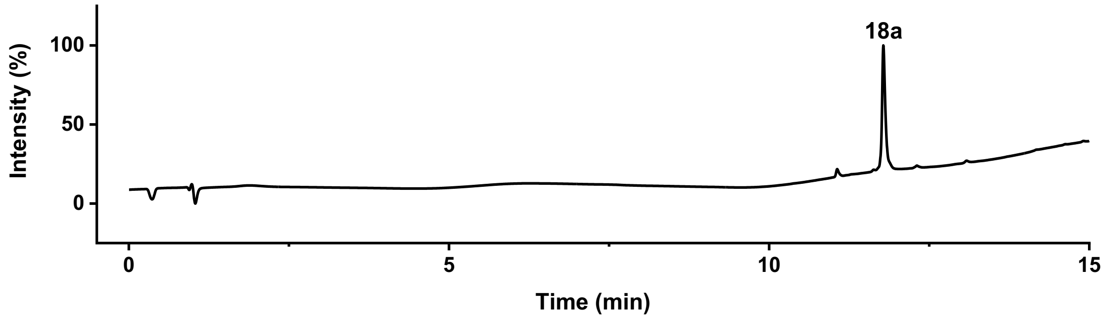


**Figure S22.** 200 nm chromatogram of purified **18a** showing linear peptide (11.78 min).

## Analytical chromatograms of *in situ* stapled peptides 1b – 18b

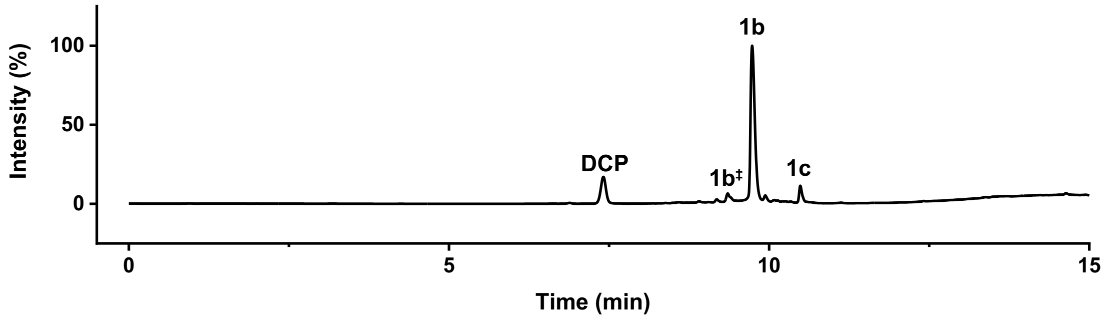


**Figure S23.** 254 nm chromatogram of crude **1b** showing DCP (7.41 min), cyclic dimer‡ (9.35 min), **1b** (9.73 min), and **1c** (10.49 min).

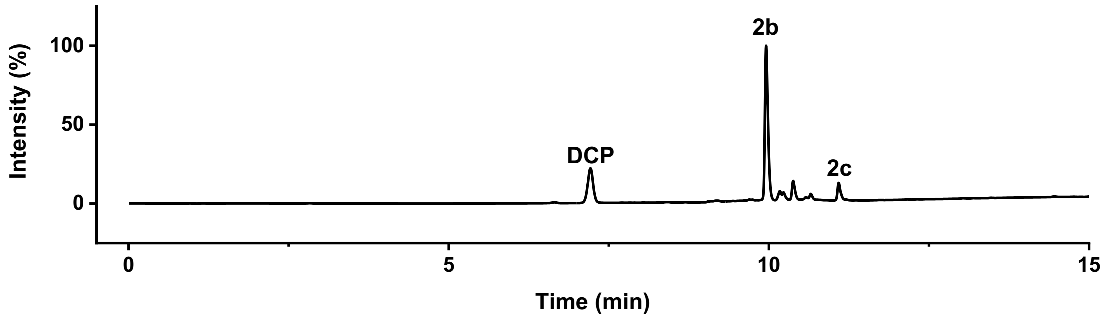


**Figure S24.** 254 nm chromatogram of crude **2b** showing DCP (7.21 min), **2b** (9.96 min), and **2c** (11.09 min).

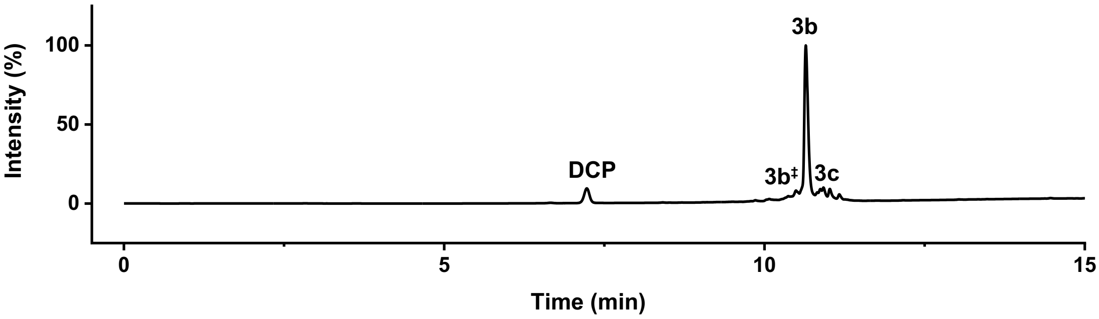


**Figure S25.** 254 nm chromatogram of crude **3b** showing DCP (7.23 min), cyclic dimer‡ (10.49 min), **3b** (10.65 min), and **3c** (11.02 min).

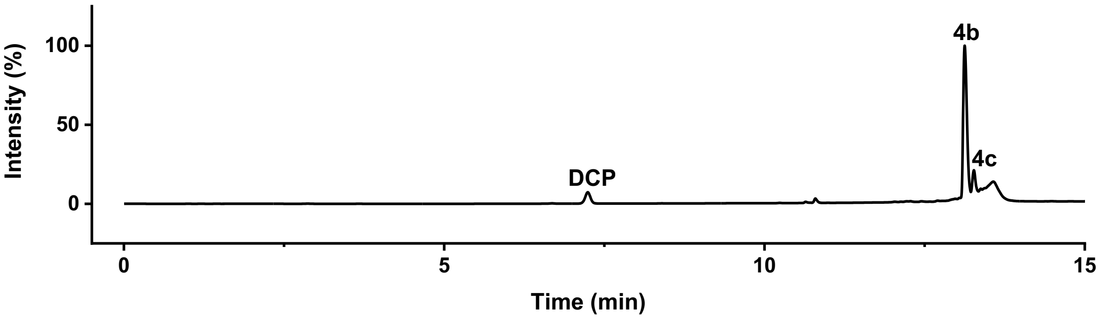


**Figure S26.** 254 nm chromatogram of crude **4b** showing DCP (7.24 min), **4b** (12.13 min), and **4c** (13.27 min).

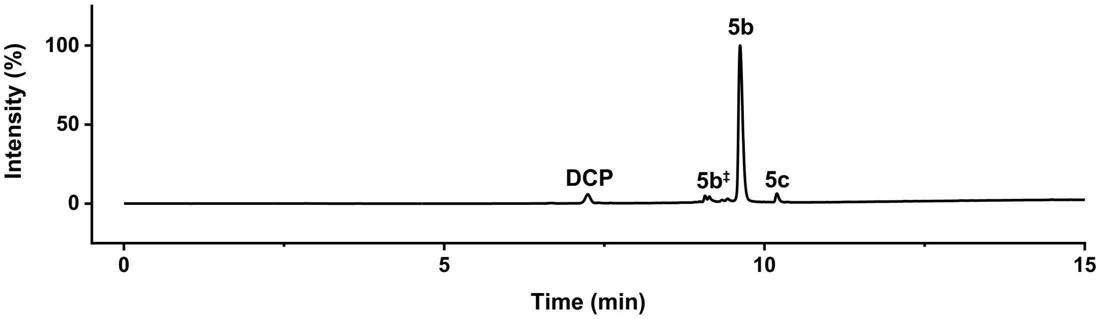


**Figure S27.** 254 nm chromatogram of crude **5b** showing DCP (7.24 min), cyclic dimer‡ (9.43 min), **5b** (9.62 min), and **5c** (10.20 min).

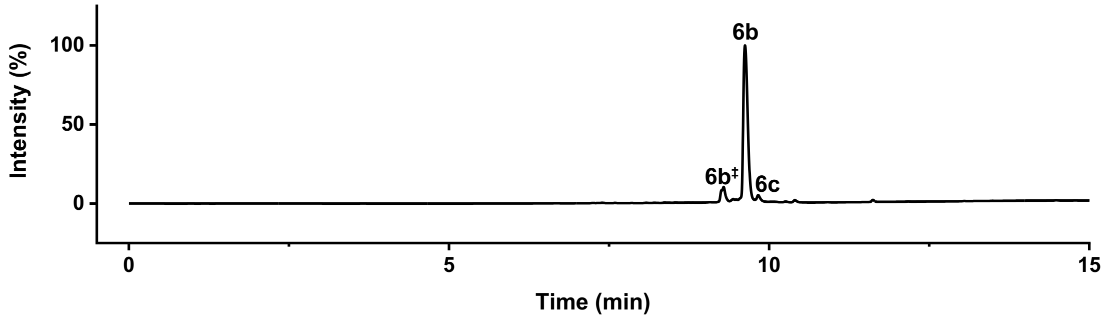


**Figure S28.** 254 nm chromatogram of crude **6b** showing cyclic dimer‡ (9.29 min), **6b** (9.62 min), and **6c** (10.40 min).

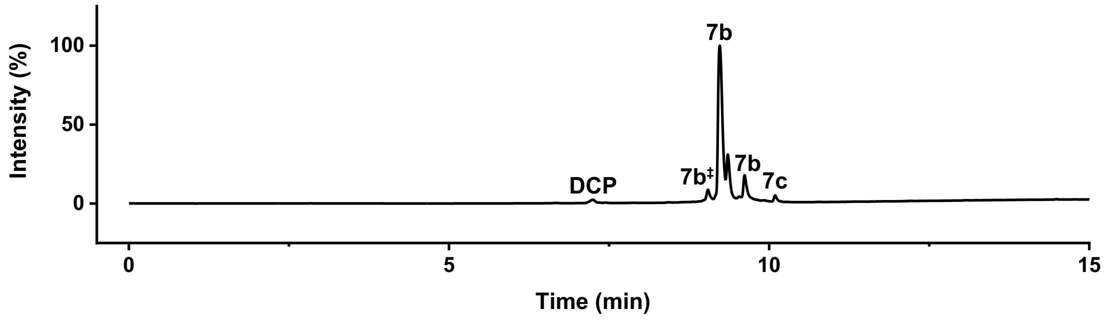


**Figure S29.** 254 nm chromatogram of crude **7b** showing DCP (7.24 min), cyclic dimer‡ (9.04 min), **7b** (9.23, 9.35, and 9.62 min), and **7c** (10.09 min).

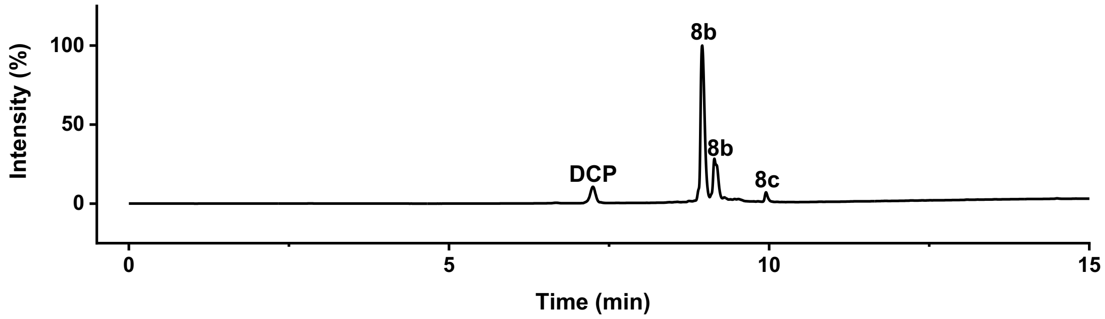


**Figure S30.** 254 nm chromatogram of crude **8b** showing DCP (7.25 min), **8b** (8.95 and 9.15 min), and **8c** (9.95 min).

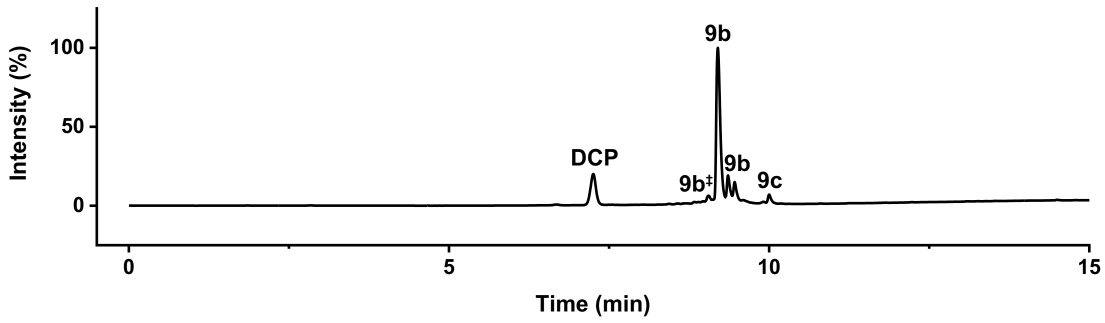


**Figure S31.** 254 nm chromatogram of crude **9b** showing DCP (7.25 min), cyclic dimer‡ (9.06 min), **9b** (9.20, 9.36, and 9.46 min), and **9c** (10.00 min).

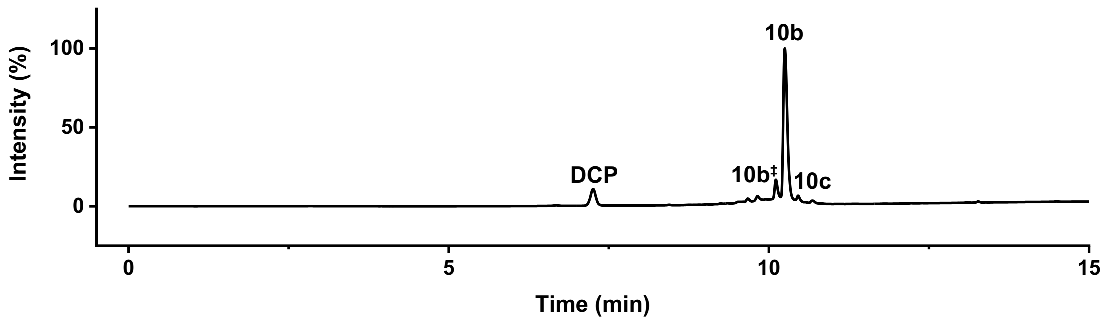


**Figure S32.** 254 nm chromatogram of crude **10b** showing DCP (7.25 min), cyclic dimer‡ (10.11 min), **10b** (10.25 min), and **10c** (10.69 min).

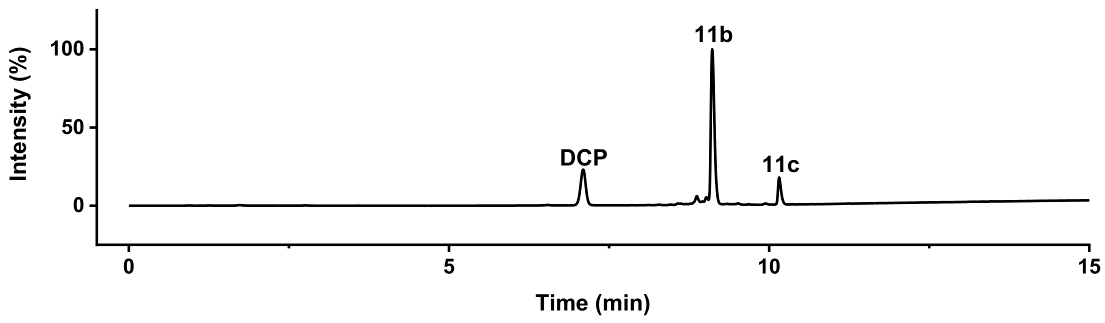


**Figure S33.** 254 nm chromatogram of crude **11b** showing DCP (7.09 min), **11b** (9.11 min), and **11c** (10.16 min).

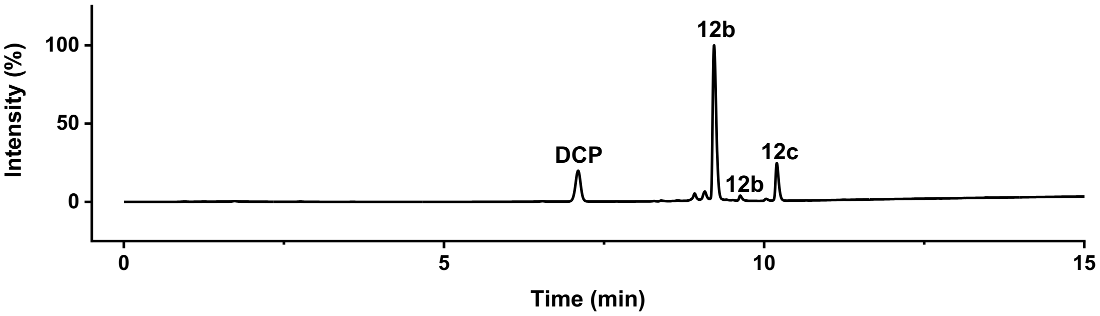


**Figure S34.** 254 nm chromatogram of crude **12b** showing DCP (7.09 min), **12b** (9.22 min and 9.63 min), and **12c** (10.20 min).

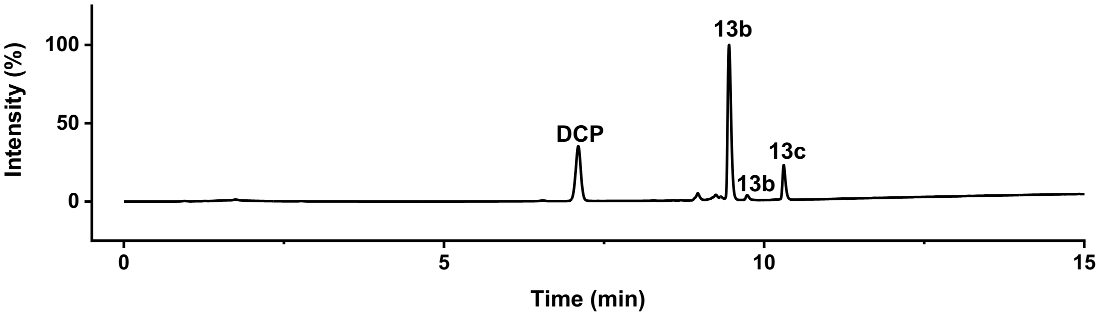


**Figure S35.** 254 nm chromatogram of crude **13b** showing DCP (7.10 min), **13b** (9.45 min and 9.74 min), and **13c** (10.30 min).

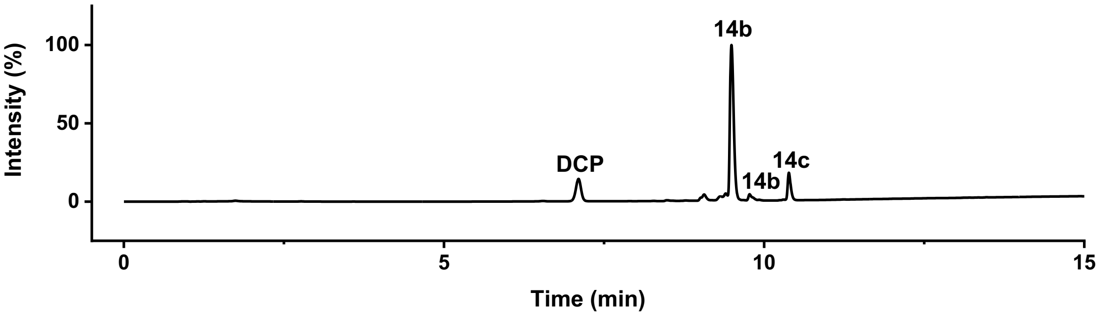


**Figure S36.** 254 nm chromatogram of crude **14b** showing DCP (7.10 min), **14b** (9.49 min and 9.77 min), and **14c** (10.39 min).

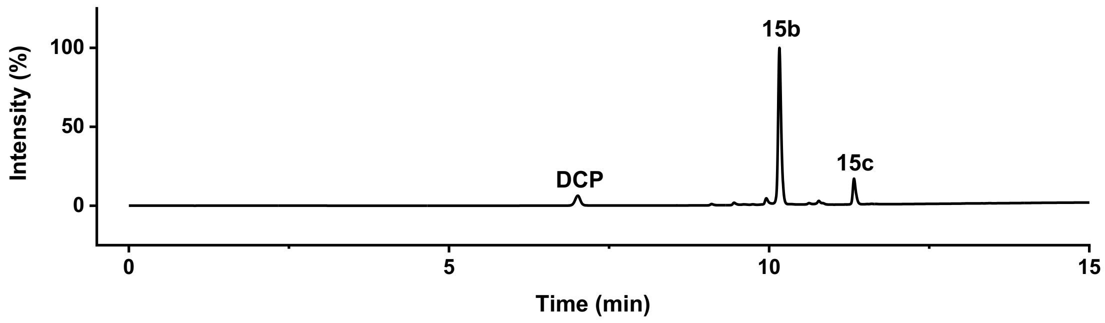


**Figure S37.** 254 nm chromatogram of crude **15b** showing DCP (7.01 min), **15b** (10.16 min), and **15c** (11.33 min).

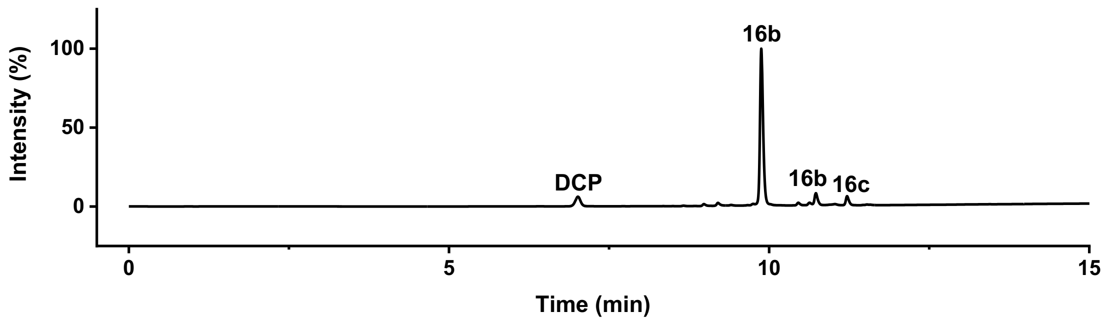


**Figure S38.** 254 nm chromatogram of crude **16b** showing DCP (7.02 min), **16b** (9.88 min and 10.73 min), and **16c** (11.22 min).

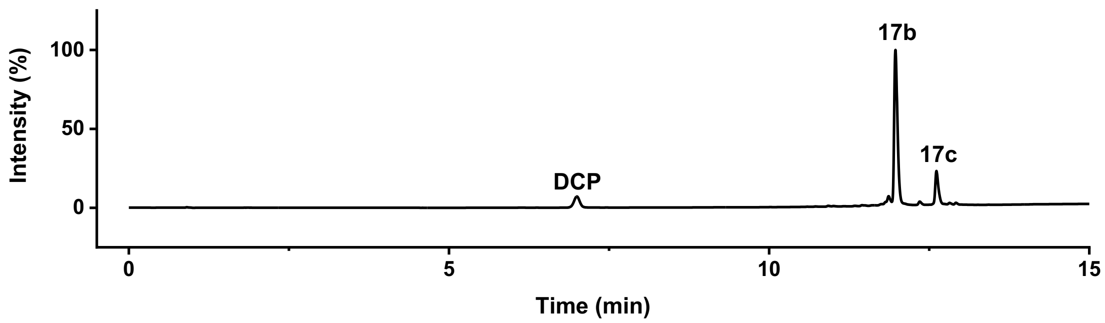


**Figure S39.** 254 nm chromatogram of crude **17b** showing DCP (7.00 min), **17b** (11.97 min), and **17c** (12.61 min).

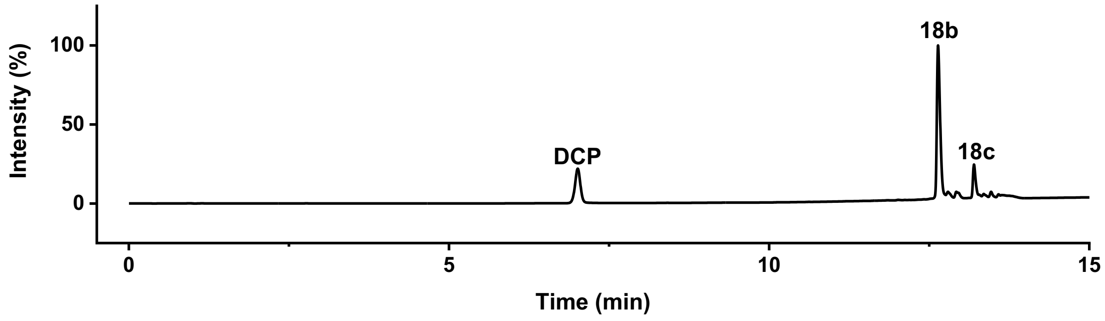


**Figure S40.** 254 nm chromatogram of crude **18b** showing DCP (7.01 min), **18b** (12.64 min), and **18c** (13.20 min). Cyclic disulfide of **18b** was detected at the identical retention time (12.64 min). High-resolution mass spectrometry (Figure S48) confirmed that the cyclic monomer is the major species and that cyclic dimer represents a negligible fraction of the peak related to **18b**.

### Analytical chromatograms of purified stapled peptides 1b and 1d

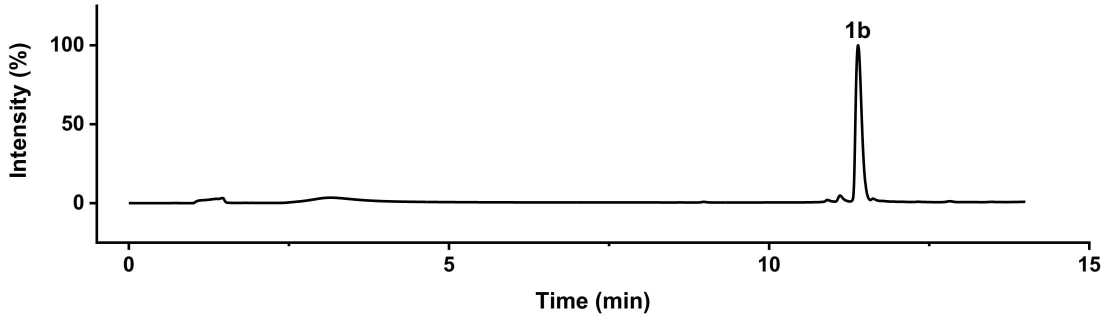


**Figure S41.** 254 nm chromatogram of purified **1b** (11.39 min).

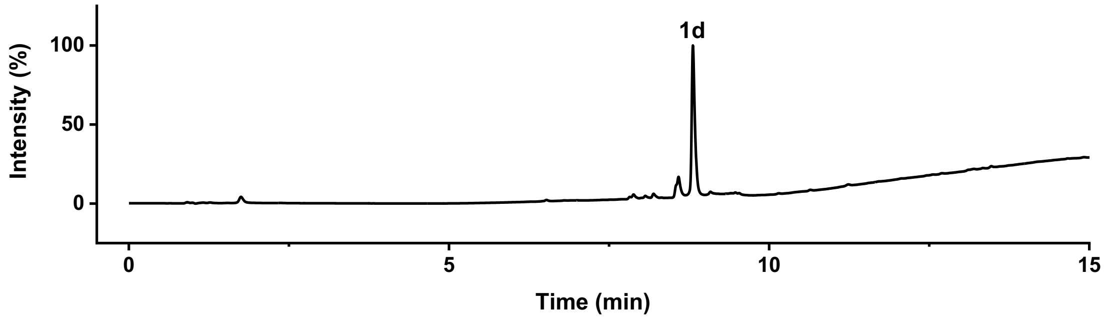


**Figure S42.** 254 nm chromatogram of purified **1d** (8.81 min).

## Analytical chromatograms of on-resin stapling experiment

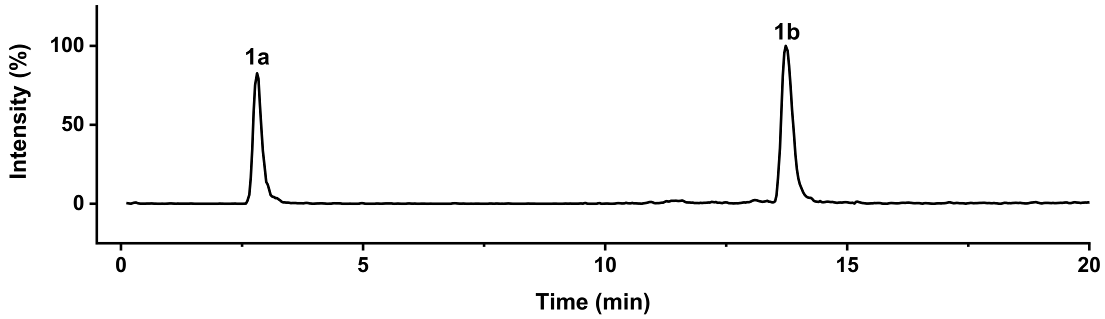


**Figure S43.** EIC of on-resin stapling using 0.5 Eq of DCP showing **1a** (linear and oxidised cyclic disulfide) (2.81 min) and **1b** (13.74 min).

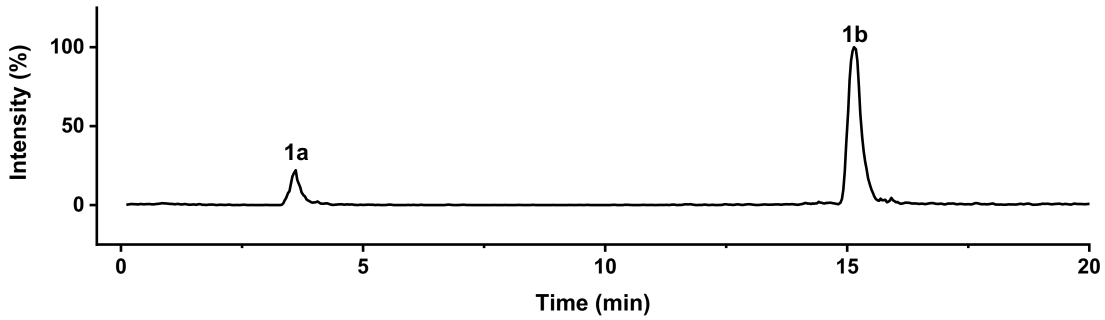


**Figure S44.** EIC of on-resin stapling using 1.0 Eq of DCP showing **1a** (linear and oxidised cyclic disulfide) (3.61 min), **1b** (15.15 min), and **1c** (15.91 min).

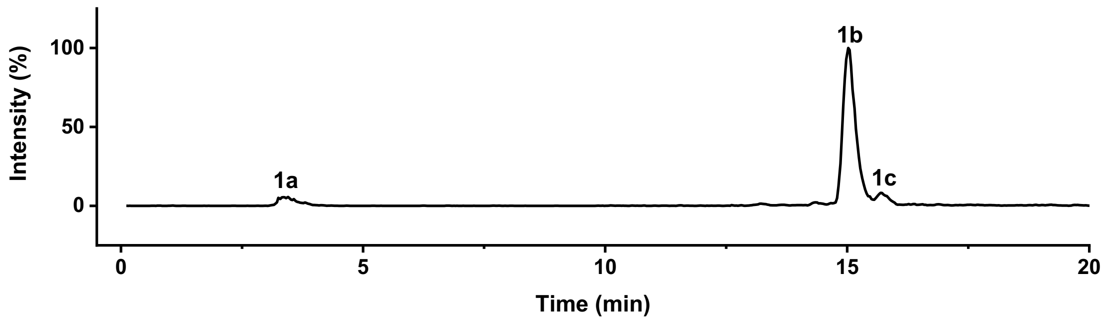


**Figure S45.** EIC of on-resin stapling using 1.5 Eq of DCP showing **1a** (linear and oxidised cyclic disulfide) (3.45 min), **1b** (15.02 min), and **1c** (15.72 min).

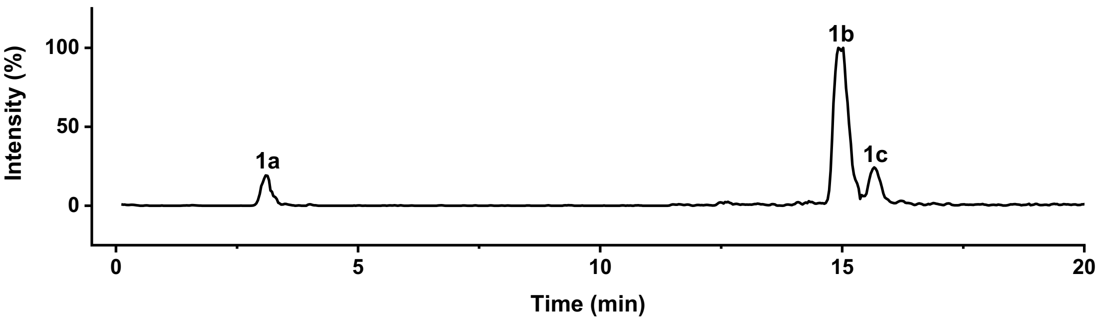


**Figure S46.** EIC of on-resin stapling using 2.0 Eq of DCP showing **1a** (linear and oxidised cyclic disulfide) (3.09 min), **1b** (14.92 min), and **1c** (15.66 min).

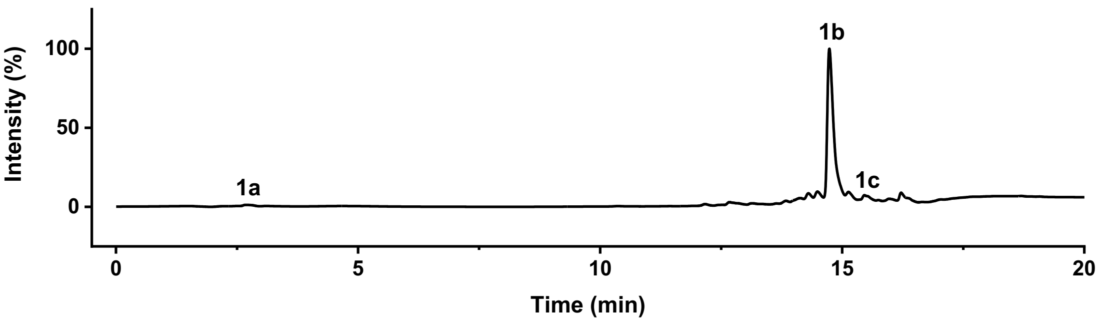


**Figure S47.** 254 nm chromatogram of on-resin stapling using 1.5 Eq of DCP showing **1b** (14.74 min) and **1c** (15.46 min).

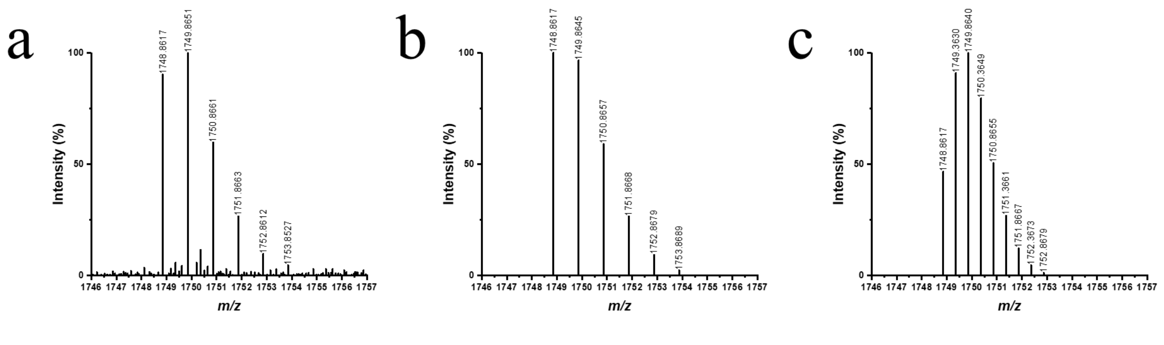


**Figure S48.** Stapling of the large peptide **18a** generates the cyclic monomer **18b** with negligible amount of cyclic dimer. a) High-resolution mass spectrum of the $[M+H]^+$ ion of **18b**. b) Calculated isotopic pattern of $[M+H]^+$ ion of **18b**. c) Calculated isotopic pattern of the $[M+2H]^{2+}$ ion of the cyclic dimer analogue of **18b**.

**Table S2.** Estimated peptide secondary structure composition.[a]

| Compound | α-Helix[b] | β-Sheet | | Turn | Others[d] | RMSD |
|---|---|---|---|---|---|---|
| | | Antiparallel[c] | Parallel | | | |
| **1a** | 0.0% | 27.5% | 0.0% | 21.5% | 51.0% | 0.139 |
| **1b** | 0.1% | 19.5% | 0.0% | 13.4% | 67.0% | 0.407 |
| **18a** | 0.5% | 24.2% | 0.0% | 19.5% | 55.9% | 0.077 |
| **18b** | 10.1% | 10.4% | 0.0% | 14.7% | 64.9% | 0.104 |

[a] Calculated from the 200 – 250 nm values of CD spectra using BeStSel web server.[2]

[b] Includes regular and distorted α-helical elements.

[c] Includes left-hand twisted, right-hand twisted, and slightly right-hand twisted β-strands.

[d] Includes $3_{10}$-helix, π-helix, β-bridge, bend, loop, and irregular structures.

## References


1 Z. Zhang, Y. Li, Y. R. Loh, W. W. Phoo, A. W. Hung, C. Kang and D. Luo, *Science*, 2016, **354**, 1597-1600.

2 (a) A. Micsonai, F. Wien, É. Bulyáki, J. Kun, É. Moussong, Y.-H. Lee, Y. Goto, M. Réfrégiers and J. Kardos, *Nucleic Acids Res.*, 2018, **46**, W315-W322; (b) A. Micsonai, F. Wien, L. Kernya, Y.-H. Lee, Y. Goto, M. Réfrégiers and J. Kardos, *Proc. Natl. Acad. Sci. U.S.A.*, 2015, **112**, E3095-E3103.
